# Sensory Traits and Consumer’s Perceived Quality of Traditional and Modern Fresh Market Tomato Varieties: A Study in Three European Countries

**DOI:** 10.3390/foods10112521

**Published:** 2021-10-21

**Authors:** Fiorella Sinesio, Maria Cammareri, Valentine Cottet, Lilian Fontanet, Michel Jost, Elisabetta Moneta, Samuela Palombieri, Marina Peparaio, Roser Romero del Castillo, Eleonora Saggia Civitelli, Patrizia Spigno, Antonella Vitiello, Brigitte Navez, Joan Casals, Mathilde Causse, Antonio Granell, Silvana Grandillo

**Affiliations:** 1 CREA—Consiglio per la Ricerca in Agricoltura e L’analisi Dell’economia Agraria—Centro di Ricerca per gli Alimenti e la Nutrizione, Via Ardeatina 546, 00178 Roma, Italy; fiorella.sinesio@crea.gov.it (F.S.); elisabetta.moneta@crea.gov.it (E.M.); marina.peparaio@crea.gov.it (M.P.); eleonora.saggiacivitelli@crea.gov.it (E.S.C.); 2 Institute of Biosciences and Bioresources, Research Division Portici, CNR—National Research Council of Italy, Via Università 133, 80055 Portici, Italy; maria.cammareri@cnr.it (M.C.); palombieri@unitus.it (S.P.); antonella.vitiello@ibbr.cnr.it (A.V.); 3 CTIFL, Centre de Saint-Rémy-de-Provence, Route de Mollégès, 13210 Saint Remy de Provence, France; valentine.cottet@ctifl.fr (V.C.); michel.jost@ctifl.fr (M.J.); brigitte.navez@wanadoo.fr (B.N.); 4 INRAE, UR1052, GAFL, Génétique et Amélioration des Fruits et Légumes, Domaine Saint Maurice, Allée des Chênes, CS 60094, CEDEX, 84143 Montfavet, France; lilian.fontanet@hmclause.com (L.F.); Mathilde.Causse@avignon.inrae.fr (M.C.); 5 Fundació Miquel Agustí-UPC BarcelonaTech, Barcelona School of Agricultural Engineering, c. Esteve Terrades, 8, 08860 Castelldefels, Spain; roser.romero.del.castillo@upc.edu (R.R.d.C.); recerca@fundaciomiquelagusti.cat (J.C.); 6 ARCA 2010 Soc. Coop. Snc, 81030 Teverola, Italy; patspigno@hotmail.com; 7 Instituto de Biología Molecular y Celular de Plantas, CSIC, Universidad Politécnica de Valencia, 46022 Valencia, Spain; agranell@ibmcp.upv.es

**Keywords:** *Solanum lycopersicum* L., landraces, fruit quality, physicochemical, sensory profile, genotype-by-environment interaction, hedonic rating, CATA

## Abstract

Consumer dissatisfaction with the flavor quality of many modern fresh market tomato varieties has fostered breeders’ interest in sensory quality improvement, and the demand for traditional varieties, which are generally associated with better flavor. To achieve further knowledge on the factors influencing the sensory quality and consumers’ preferences and perception, European traditional and modern fresh market tomato varieties were grown and evaluated in France, Italy, and Spain. Different growing conditions were tested in France (soilless vs. soil) and in Spain (open field vs. greenhouse), while in Italy fruits were evaluated at two ripening stages. Fruit quality was assessed by integrating physicochemical analyses, sensory profiles, and consumer tests. In all three countries, overall modern varieties were perceived as having more intense “tomato flavor” and “overall flavor” than traditional ones. In France and Spain, consumers’ preferences were more oriented towards modern varieties than traditional ones. Significant growing condition effects were found on sensory and physicochemical traits, while the effect on consumers’ overall liking was not significant, largely depending on the genotype. A fair agreement between product configurations from descriptive analysis by trained assessors and Check-All-That-Apply (CATA) questions by consumers was observed. Penalty-lift analysis based on CATA allowed identifying positive and negative drivers of liking.

## 1. Introduction

Tomato (*Solanum lycopersicum* L.) is one of the most widely grown and frequently consumed crops worldwide (www.fao.org/fastat (accessed on 4 March 2021)), representing an important source of essential nutrients and nutraceutical compounds to the human diet [1].

Tomato fruit quality for fresh consumption depends on numerous traits relating to external (appearance, hand-evaluated texture), and internal (texture, flavor and nutritive value) attributes, most of which are polygenic traits influenced by the genotype, the environment, the agronomic practices, but also the maturity stage [2,3,4]. Considering the purchase environment, external quality attributes are primary drivers in the initial consumer’s choice [5], while eating quality becomes the major influencing factor in subsequent purchases [6]. Preference mapping studies conducted on fresh market tomatoes have frequently reported flavor and texture to be the main factors driving and differentiating consumer preferences [7,8,9,10].

Flavor, which denotes a combination of taste and retronasal olfaction, is a particularly complex phenotype, influenced by many objective and subjective factors, and that involves multiple sensory systems [11]. The characteristic sweet-sour taste of tomato and its overall flavor intensity is chemically determined by a complex blend of interplaying primary and secondary metabolites mainly comprising of sugars (glucose and fructose), acids (citrate, malate and glutamate), minerals and multiple key volatile compounds [12,13,14]. However, texture, which is also a complex trait including several components [15], appearance, temperature, mouthfeel and previous experience, also impacts the whole sensory quality and influence flavor and odor perception [12,13].

Despite tomato’s versatility and popularity, over the last three decades, the flavor of most commercial tomato varieties has been a major source of consumer complaint [11,16]. Domestication and modern breeding efforts primarily focusing on improving growers’ targets such as fruit size, yield, shelf life, resistance to pests and diseases, along with commercial harvesting of immature fruits and storage practices, have had detrimental effects on the tomato flavor [17]. Furthermore, for several years, flavor has been a largely neglected target, mainly due to the difficulty in selecting such a complex phenotype in the absence of efficient objective selection criteria [4,11]. To reverse this trend, and to meet consumers’ demand, sensory quality has become another key objective for tomato breeders [17,18]. In this context, “heirloom” and local or traditional tomato varieties (landraces), adapted to specific agro-climatic conditions, are gaining popularity among consumers, as they are generally considered to have a superior flavor (“flavor of the past”), but also for their cultural link with a territory [11,19]. This has fostered research programs aimed at investigating the genetic and phenotypic variability of these important genetic resources [18,19,20,21,22], but also at studying their sensory profiles [23,24,25,26], and, to a lesser extent, at identifying the key instrumental traits and sensory descriptors that meet consumers’ preferences [14,27]. For the US tomato consumers’ market, the flavor breeding targets have been largely defined by exploring the diversity of a large panel of modern, heirloom and wild tomato accessions [14]. However, consumers’ preferences may vary among countries, and each market segment might have different target compositions. Additionally, the comparisons between commercial modern and traditional varieties may lead to different conclusions also depending on the specific set of genotypes used in the study [18]. Given the important role of environmental and genotype × environment interaction effects on tomato fruit sensory quality [7,28,29,30], site sensory variation should also be explored by testing the influence of the different growing conditions on consumers’ preferences and perceptions.

Traditionally, the approach used to study the relationship between sensory properties and consumer preferences combines classical descriptive sensory analysis (DA) by trained panels with hedonic measures by consumer panels [31]. In recognition of the importance of developing products that meet consumers’ demands and assure acceptance, during the last decade several novel consumer-based sensory characterization approaches have been developed, and Check-All-That-Apply (CATA) questions are one of the most popular [32]. This method consists of presenting respondents with a list of pre-determined words or phrases and asking them to check all the terms they consider appropriate to describe the test product [32]. As no measure of intensity is required, CATA questions are regarded as easy to answer for consumers; nevertheless, the method has a high discriminative capability among test samples and produces results similar to those obtained from DA by trained panels [32,33,34,35,36].

Building on these premises, the main objectives of this study were: (i) to assess the sensory quality of European traditional fresh market tomato varieties, belonging to different typologies, compared to their modern counterparts; (ii) to study the effect of different growing conditions on physicochemical properties, sensory profiles, consumers’ preferences and perceptions in three European countries; (iii) to compare sensory product configurations generated by trained assessors (DA) and consumers using CATA questions, and to identify potential drivers of liking for fresh market tomatoes based on consumers’ perception.

## 2. Materials and Methods

### 2.1. Plant Material and Growing Conditions

Seventeen traditional and modern fresh market tomato varieties belonging to the typologies “Oxheart”, “Oxheart Liguria”, “Marmande” and “Round” were selected for this study, and different partially overlapping sets were grown and evaluated in France, Italy and Spain during spring/summer 2016 (Table 1).

In France, 12 varieties were grown simultaneously in two locations: soilless (HS) cultivation, in glasshouses at the CTIFL in Balandran center (near Nimes) and in soil (S), under a plastic tunnel at INRA in Avignon. Fruits were harvested at stages C5 to C7, following the 12-sheet OCDE color code distributed by CTIFL except for Garance and HybridInra (H13.91) which were harvested at stage C9 in accordance with the type of fruit.

In Spain, 11 varieties were grown in the locality of Palafolls, using two different growing environments concurrently: greenhouse (GH) and open field (OF). Fruits were harvested at stages C6 to C8.

In Italy, 12 varieties were grown in soil under a tunnel-greenhouse on a farm in Acerra (Naples). Fruits were harvested at two ripening stages: “early ripening” (ER) (stages C3 and C4) and RR (stages C6 to C8).

In each country, upon harvest, fruits were divided into homogenous batches reserved for sensory analyses and for physicochemical measurements. Fruits were stored under a controlled temperature 14 °C ±2 °C and were used within 2 days after harvesting. Selected subsets of products were used for the consumer tests (Table 1).

### 2.2. Physicochemical Analyses

Nondestructive physical measures were performed on a single-fruit basis: fruit weight (FW); fruit firmness (FIRM), measured with durometers Durofel: SETOP (nozzle 0.25 cm^2^) in France, Agrosta 100 Field in Spain, and AGROSTA^®^100 USB in Italy; and external fruit color (EC) determined using the CIE L*a*b* color space, with Konica Minolta chromatometers (EC_L*, EC_a*, EC_b*). FIRM and EC were both measured on two diametrically opposite sides of the equatorial plane of the fruit.

Chemical measures were taken on the supernatant of centrifuged blended tomato juice stored at −25 °C until analysis. For each replicate, 5–10 fruits were cut and then homogenized. The soluble solids content (SSC) was evaluated with refractometers: an Atago pallets PR32 in France, a handheld ERMA (0–18%) in Spain, and ATAGO (PAL-1) in Italy. Titratable acidity (TA) was measured by potentiometric titration of the sample using 0.1 M NaOH (titration to pH 8.1). In France, sugars (sucrose, fructose and glucose) and acids (citric and malic acids) were measured by High Performance Liquid Chromatography (HPLC). In Spain, blended tomato juice was used for the dry matter (DM) (65 °C) measurements.

### 2.3. Sensory Analysis

#### 2.3.1. Descriptive Analysis (DA)

DA took place in sensory laboratories conforming to international standards for laboratories approved for sensory analysis [37], under white light, at a temperature of 22 °C ± 2 °C. The samples were removed from the stalk and crown, washed with cold running water, and dried with a paper towel. A whole tomato was served per sample on a disposable plate. The products were presented as a blind test (identified by codes with 3 random numbers), in a monadic mode, and in a complete balanced experimental plan. Varieties or growing conditions were assigned to each session at random. The assessors were provided with a kitchen knife, and with mineral water, unsalted breadsticks or crackers to rinse their mouth before the first and between each sample.

Highly trained sensory panels were composed of 14 assessors in France, 8 in Italy, and 9 in Spain, specifically trained in the sensory evaluation of fresh tomato [8,9,38]. In each country, 2–3 pre-testing sessions were run to reach a consensus on the list of sensory descriptors and for calibration.

Nine attributes were common to the three countries, but specific descriptors familiar to the panels were added in France and Italy (Table 2). Each product was tasted twice by each panelist, and each panel adopted their standard evaluation scale (0 to 10 discontinuous, in France, and semi-structured continuous, in Spain; 0 to 9 continuous, in Italy), anchored at the extremes as “null” and “high” perception.

In France, the sensory panel used a lexicon of 15 descriptors to taste 48 samples in 9 sessions held at the Balandran CTIFL sensory analysis laboratory.

In Spain, the sensory panel used the 9 common sensory attributes to evaluate 44 samples in 11 sessions held at the laboratories of the Barcelona School of Agricultural Engineering—UPC.

In Italy, 13 descriptors were used to taste 48 samples in 8 sessions that took place at the CREA sensory analysis laboratory in Rome. The first 4 sessions were used to evaluate the 24 ER samples and the following 4 sessions for the 24 RR samples.

For all three panels, a maximum of two sessions were held per day, in the morning, with an average duration of 30–45 min.

#### 2.3.2. Consumer Tests

In parallel to the DA, consumer tests were conducted in one large city per country (Paris, Barcelona and Naples), in order to target the same type of population in the three countries. The subset of varieties and growing conditions/ripening stages used for the consumer tests are given in Table 1.

Voluntary participants had to be regular consumers of fresh tomatoes (at least twice a month) and equilibrated in gender, and age (33% of classes 18–34 y, 35–49 y and over 50 y, with maximum 10% over 65 y) in order to have a representative sample of the entire population. They were not aware of the objective of the study and did not receive any information on the products. All subjects gave their informed consent to participate in the study, which was approved by the CNR Research Ethics and Integrity Committee (IRB, Prot. N. 0052403/21). Personal data were protected by anonymization according to National Data Protection Regulations. The tests were run in a neutral environment according to ISO 11136 [39].

In France, 103 consumers were recruited by a service provider in Paris. The tests were conducted on 14 products, 7 cultivated in HS and 7 in S. Each consumer participated in two sessions one week apart.

In Spain, the panel was composed of 133 consumers, recruited by the sensory lab of the Barcelona School of Agricultural Engineering. The tests were conducted on 16 products, 10 cultivated in GH and 6 in OF, during 2 sessions, held over 2 consecutive days.

In Italy, 107 consumers were recruited by a service provider in Naples. The tests were conducted on 20 products, 12 were tested at the ER stage and 8 at the RR stage. Each consumer participated in 4 sessions on different days, two sessions for tomatoes at ER stage and the following two for tomatoes at the RR stage. 

In all three countries, tomato samples were presented in a monadic sequential, according to a balanced design (Williams Latin square design) to avoid effects of order and first position. No dummy sample was presented as a first product. Each sample was presented in a disposable plate blinded by a three-digit randomized code. Consumers were provided with water to cleanse the palate between samples. Overall liking (OL) evaluation in France and Italy was done on a nine-point hedonic scale anchored with 1 (“dislike extremely”) and 9 (“like extremely”) and in Spain on a 1 to 10 scale (same anchors).

In addition to the global appreciation, consumers were asked to answer a Check-All-That-Apply (CATA) questionnaire to describe the consumers’ perception of each test product. The consumers could check up to 20 (France and Spain) and 24 (Italy) terms relating to visual aspect, texture and flavor, which were selected from the literature (Table 2). In Italy, three terms for external color were added (“too green”, “too red”, “too pale color”), as the tomato varieties were evaluated at two different ripening stages. Overall, 19 terms were common to the three countries. As recommended by Ares and Jaeger [40], the order of the CATA terms was randomized for each assessor.

### 2.4. Statistical Analysis

The data obtained from each experiment were analyzed through the Analysis of Variance (ANOVA) considering genotype (G) and growing condition (GC) as sources of variation and interaction. ANOVA was also used to test varietal group (Traditional/Modern) and G × GC interaction effects. Tukey’s Honestly Significant Difference (HSD) multiple comparison procedure (*p* ≤ 0.05) was performed to check which of the evaluated varieties and GCs were distinguishable in sensory, physicochemical, and hedonic dimensions. Principal Component Analysis (PCA) was used to envisage sample groups based on sensory and physicochemical data. PCA based on Pearson Correlation coefficients was carried out on normalized data.

The frequency of use of each CATA term was determined and the non-parametric Cochran’s Q test was carried out on the raw binary CATA data to identify which sensory terms were significantly discriminating among samples. Multiple pairwise comparisons were based on McNemar’s test. The relationship between samples and CATA terms was evaluated by means of correspondence analysis (CA), performed on the frequency table from each experiment, considering chi-square distances. To identify the terms that lead to a lower or a higher OL score, penalty-lift analysis was carried out on the CATA data of each country as proposed by Meyners [41]. This analysis determines the drop in OL associated with a deviation for each CATA term. Only terms mentioned with a frequency > 20% have been included in this analysis.

Similarity configuration between DA and CATA was assessed by Multiple Factor Analysis (MFA) on the common samples. For the MFA analysis, one matrix was constructed using the frequency matrices corresponding to CATA and one matrix with average intensity ratings for the DA. A quantitative measure of proximity between sample configurations was obtained by Regression Vector (RV) coefficient [42]. RV values range between 0 and 1, with 1 indicating the highest similarity between configurations obtained by the two data matrices.

All the statistical analyses were carried out using the XLStat software (v. 19.4, copyright Addinsoft 1995–2019). For all the analyses the significance level was set at *p* ≤ 0.05.

## 3. Results

### 3.1. Experiment in France

#### 3.1.1. Descriptive Analysis and Physicochemical Data

In France, ANOVA revealed a significant varietal group (Modern/Traditional) effect on all sensory descriptors, except for “rib aspect” and “sweet taste” (Table S1). Regarding appearance, altogether, traditional varieties were perceived as larger and less colorful than the modern ones. The latter were perceived as firmer, crunchier, less melting, and less mealy, but juicier, with thicker skin, more acidic and with more pronounced “overall odor”, “overall flavor” and “tomato flavor”. A significant GC effect was observed for 11 descriptors, and, altogether, varieties cultivated in S were perceived as smaller, less firm, less crunchy, and less mealy, but juicier and meltier than HS products. Regarding flavor descriptors, S products were perceived as more acidic, with more pronounced “overall odor”, “overall flavor” and “tomato flavor” than HS products. The genotypic effect was highly significant for all the descriptors, and a significant G × GC interaction was observed for 10 of the 15 descriptors.

With respect to the physicochemical parameters, ANOVA showed statistically significant effects of both the varietal group and GC (Table S2). Overall, SSC, TA, glucose, fructose, citric acid, and FIRM were higher in the modern variety group, while FW, b*, and malic acid were higher in the traditional one. The different GCs significantly influenced all parameters but FW, with higher values observed for the S cultivation, except for FIRM. Genotypic and G × GC effects were highly significant for all parameters, except for the G × GC effect on SSC, which was significant at *p* < 0.05.

**Figure 1:**
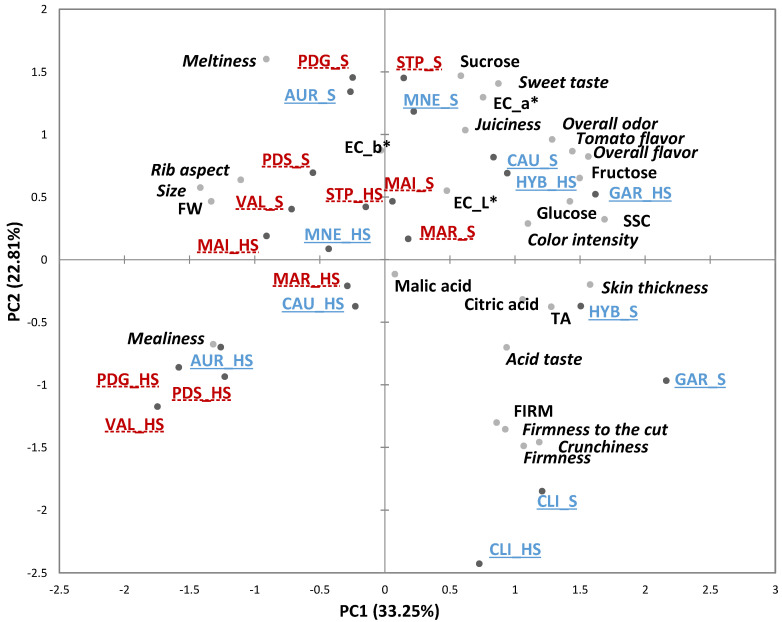
Principal component analysis (PCA) Bi-plot of the sensory and physicochemical parameters of the 12 tomato genotypes grown in Soil (S) and Soilless (HS) in France. Sensory descriptors are indicated in *italics*. Modern varieties are in blue, underlined with solid lines; Traditional varieties are in red, underlined with dashed lines. FW = fruit weight, FIRM = firmness (instrumental), SSC = soluble solids content, TA = titratable acidity. Product codes are listed in Table 1.

The PCA plot based on sensory and physicochemical data is shown in Figure 1. The first two principal components (PCs) accounted for 56.06% of the total variance. The variables that most contributed to the variance explained by the negative semi-axis of PC1 were “size”, “ribbed aspect”, “mealiness”, FW, while for the positive semiaxis were “color intensity”, “skin thickness”, “overall odor” and “overall flavor”, “tomato flavor”, glucose, fructose, SSC, and TA. The projection, on the PCA plot, of the 12 varieties grown in the S and HS conditions did not reveal any clear separation of the products according to the growing environment. However, for all 12 varieties, HS products had lower values for the PC1, with most of the “Oxheart” and “Marmande” HS tomatoes gathering on the negative side of PC1. In contrast, the three modern round varieties (*GAR*, *CLI* and *HYB*, cultivated in S and HS), all located on the positive semi-axis 1, were perceived as colorful, firm, crunchy, with a thick skin and flavorful. The other varieties (traditional “Round”, and some of the modern and traditional “Oxheart” and “Marmande” typologies), most of which were grown in S, are plotted on the positive semi-axis 2. A major contribution to this dimension is given by the variables “meltiness”, “sweet taste”, sucrose, EC_a*, on the positive side, and “firmness”, “firmness to cut”, “crunchiness” and FIRM, on the negative side.

#### 3.1.2. Consumer Data

The average overall liking (OL) values of the 14 tomato products evaluated in France under the two GCs ranged between 5.2 and 6.6/9, showing that all products were appreciated, and none rejected (Table 3). ANOVA highlighted a highly significant varietal group (Modern/Traditional) effect on OL with acceptance of modern varieties slightly higher than for the traditional ones. The genotype was also a highly significant factor, while, overall, the two different GCs did not have any significant effect on OL, although a significant G × GC effect was observed. Considering both growing systems, *MNE* seemed to be a favorite genotype among the modern varieties, although the difference was not statistically significant, and *STP* was preferred among the traditional ones.

With respect to the CATA data, Table S3 shows the frequency with which each of the 20 terms was mentioned by the French consumers to describe the tomatoes under study. The nine most frequently cited terms (mean occurrence > 20%) were: “beautiful color”, “I like its appearance”, “I like its fleshy aspect”, “juicy”, “pleasant aroma”, “aromatic/strong aroma”, “too thick skin”, “not enough sweet”, “bland/no taste”. The non-parametric Cochran’s Q test revealed highly significant (*p* < 0.001) differences in the frequencies of citing for all the CATA terms presented to the consumers (Table S3). A bi-dimensional map derived from the Correspondence Analysis (CA) run on the contingency table of the CATA terms explained 60.86% of the inertia (Supplementary Materials Figure S1). Among the most liked samples, *MNE*_S and HS, *CAU*_S and HS, and *STP*_S were perceived by consumers as “aromatic”, “fleshy” and “juicy”. By contrast, among the least liked samples, *MAI*_S and HS were perceived as “hollow” and “mealy”, with *MAI*_HS being also perceived as “too soft”, while *VAL*_S was perceived as “mealy” and “too soft”. In addition, all the three least liked samples were also perceived as “not enough sweet” and with a “bland/no taste”. Most of the Round tomato samples (*GAR*_S and HS, *CLI*_HS and *STP*_HS) were associated with “beautiful color” and a “liked appearance”. However, the three Modern Round samples (*GAR*_S and HS, *CLI*_HS) were also perceived as with a “too thick skin”, and *CLI*_HS and *GAR*_S also as “too firm”.

The penalty-lift analysis identified “aromatic” (1.926), “strong aroma” (1.646), “juiciness” (1.254), “fleshy aspect” (1.238), “liking of appearance” (1.185) and “beautiful color” (1.126) as potential higher positive directions for OL’s improvement and “bland taste” (−1.827), “not enough sweet” (−1.141) and “too thick skin” (−0.788) as the main negative attributes.

The relationship between consumers’ and trained assessors’ sensory profiles was evaluated via a comparative MFA on the output of both the CATA and DA data. The analysis showed that the two product configurations were comparable, with an RV coefficient of 0.78, and the superimposed representation of the samples in the MFA allows the evaluation of their proximity for each sample (Figure S2a). Many terms were comparable and well correlated, with good associations found for taste (sweet, acid), flavor and texture (firmness, juiciness, mealiness, skin thickness), with significant (*p* < 0.05) correlation coefficients ranging from a minimum of r = 0.55 for “mealiness” to a maximum of r = 0.89 for “juiciness” (data not shown).

### 3.2. Experiment in Spain

#### 3.2.1. Descriptive Analysis and Physicochemical Data

In Spain, a highly significant varietal group (Modern/Traditional) effect resulted from the ANOVA for the sensory descriptors “sweet taste”, “overall flavor”, and “tomato flavor” (Table S4). Altogether, modern varieties were perceived as sweeter and with more intense “overall flavor” and “tomato flavor”. The GC effect was significant for “overall odor”, “acid taste” and “firmness”, with higher values observed in varieties grown in GH, and for “mealiness”, which was higher in OF products. The genotypic effect was significant for all the descriptors, while a significant G × GC interaction was detected for “sweet taste”, “overall flavor”, “tomato flavor” and “mealiness”. In addition, ANOVA showed a significant varietal group effect on five physicochemical parameters (Table S5). Specifically, SSC, TA and DM were higher in the modern variety group, while b* and pH were higher in the traditional one. The GCs significantly influenced only SSC and FIRM with higher values observed in the OF cultivation. The genotypic effect was highly significant in all cases, whereas the G × GC effect was significant for five of the eight measured parameters, including all the color parameters, FIRM and DM.

**Figure 2:**
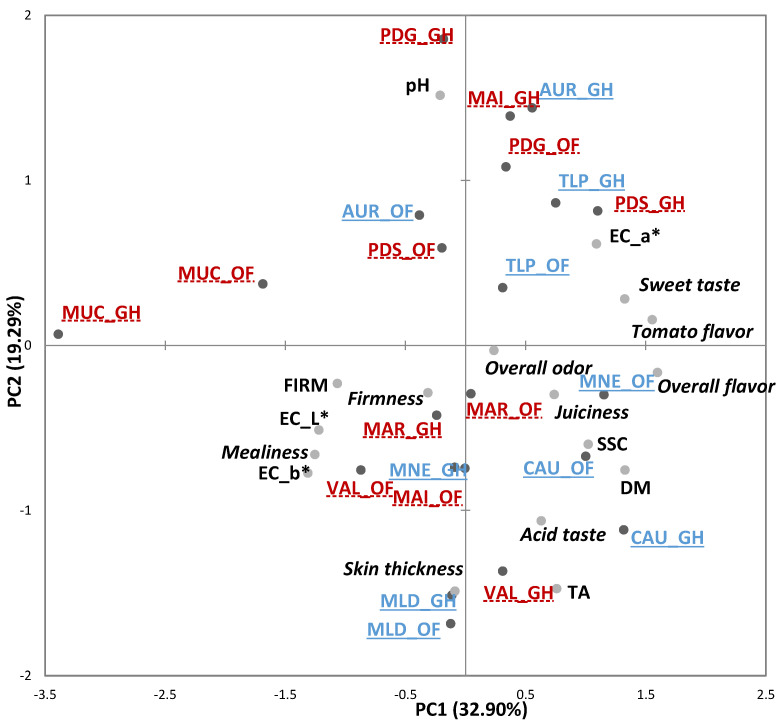
Principal component analysis (PCA) Bi-plot of the sensory and physicochemical parameters of the 11 tomato genotypes grown in Greenhouse (GH) and Open Field (OF) in Spain. Sensory descriptors are indicated in *italics*. Modern varieties are in blue, underlined with solid lines; Traditional varieties are in red, underlined with dashed lines. FIRM = firmness (instrumental), SSC = soluble solids content, TA = titratable acidity, DM = dry matter. Product codes are listed in Table 1.

The first two PCs of the PCA accounted for 51.19% of the variability among the sensory attributes and the physicochemical parameters (Figure 2). As in the French study, the projection of the 11 varieties grown in the OF and GH conditions did not reveal any clear separation of the products according to the growing system. The variables that most contributed to the variance explained by the PC1 were “sweet taste”, “overall favor”, “tomato flavor”, SSC, DM, a* on the positive side and “mealiness”, FIRM, EC_L*, EC_b*, on the negative side. The modern varieties *MNE*_OF and *CAU*_OF and *CAU*_GH, along with the traditional variety *PDS*_GH, showed higher positive coordinates along this dimension. In contrast, *MUC*_GH and *MUC*_OF, with more extreme positions on the negative semi-axis of PC1, were perceived as “mealy”, low in “tomato flavor” and “sweet taste”, with high EC_L*, EC_b* and FIRM. *PDG*, *TLP*, and *AUR*, cultivated in GH and OF, *MAI*_GH and *PDS*_OF, plotted on the positive semi-axis of PC2, were tendentially perceived as less acid. *MLD*_GH and OF, *VAL*_GH distinguished for having a thicker skin. All the other samples had intermediate characteristics.

#### 3.2.2. Consumer Data

The average OL values of the 16 tomato products tested in Spain under the two GCs ranged between 4.4 and 6.8/10, showing that all products, but the traditional variety *MUC*, were appreciated (Table 4). ANOVA showed highly significant varietal group (Modern/Traditional) and genotypic effects on OL with higher acceptance of modern varieties with respect to traditional ones, except for the landrace *PDS*. Conversely, overall, the GC effect was not significant, although, a significant G × GC interaction was found.

With respect to the CATA questions, similar to the results obtained in France, the most frequently cited terms (mean occurrence > 20%) were: “beautiful color”, “I like its appearance”, “I like its fleshy aspect”, “juicy”, “too thick skin”, “not enough sweet”, “bland/no taste”, along with “sweet” that was not included in the list of terms used in France. According to Cochran’s Q test, highly significant (*p* < 0.001) differences in the frequencies of citing were observed for all the CATA terms (Table S6). The first two dimensions of the CA restituted 63.11% of the inertia (Figure S3). The map shows that the most liked tomato products (*PDS*_GH, *MLD*_GH and OF, *AUR*_GH, *CAU*_GH and OF, *MNE*_GH and OF) were perceived by consumers as “sweet”, “juicy”, with a “fleshy aspect”, a beautiful “color” and “liking of appearance”, while the least liked one, *MUC*_GH, was perceived as “too firm”, with “mealy” texture, “too thick skin”, “unpleasant appearance”, “not enough sweet” and “tasteless”.

The penalty-lift analysis highlighted “sweet tomato” (1.576), “fleshy aspect” (1.357) “juiciness” (1.201), “beautiful color” (1.096) and “appearance” (0.970) as potential higher positive directions for OL’s improvement and “bland/no taste” (−1.972), “not enough sweet” (−0.970), and “too thick skin” (−0.775) as the main negative attributes.

MFA analysis revealed a fairly good agreement between DA and CATA configurations (RV = 0.68) (Figure S2b). Among the comparable descriptors and CATA terms, good correlations were found for taste (sweet, acid), flavor and texture (firmness, juiciness, skin thickness), with statistically significant (*p* < 0.05) correlation coefficients ranging from a minimum of r = 0.52 for “overall flavor” to a maximum of r = 0.81 for “sweet taste” (data not shown).

### 3.3. Experiment in Italy

#### 3.3.1. Descriptive Analysis and Physicochemical Data

Descriptive profiling and physicochemical characterization were performed in Italy on 12 cultivars at early ripening (ER) and red ripe (RR) stages (Table 1).

At the ER stage, ANOVA runs on the sensory descriptors showed a significant effect of varietal group (Modern/Traditional) on “external color”, “overall flavor”, “tomato flavor”, “firmness” and “juiciness” (Table S7). Specifically, modern varieties were perceived as more red-colored, juicier, and with more pronounced “overall flavor” and “tomato flavor”, whereas landraces were perceived as firmer. In addition, a highly significant genotypic effect was observed on “seeds”, “pulpiness”, “sweet taste”, “acid taste” and “juiciness”, while significant effects were also found on “external color” and “tomato flavor”. As regards the physicochemical parameters, a highly statistically significant varietal group effect was observed only for pH and TA (Table S8), and overall, modern varieties were more acid. Conversely, a highly significant genotypic effect was found for all the parameters, except for EC_a* and FIRM.

At the RR stage, ANOVA revealed a significant effect of the varietal group on the sensory descriptors “overall odor”, “sweet taste” and “overall flavor” (Table S9). Altogether, modern varieties were perceived as sweeter and with more pronounced “overall odor” and “overall flavor”. Furthermore, a significant genotypic effect was observed for the descriptors “external color”, “seeds”, “overall odor”, “sweet taste”, “acid taste” and “firmness”.

As regards the physicochemical parameters, a highly significant varietal group effect was observed for EC_a*, SSC, pH and TA (Table S10), and altogether modern varieties had higher TA, SSC, and EC_a* values. At the RR stage, the genotypic effect was highly significant for all the analyzed parameters.

**Figure 3:**
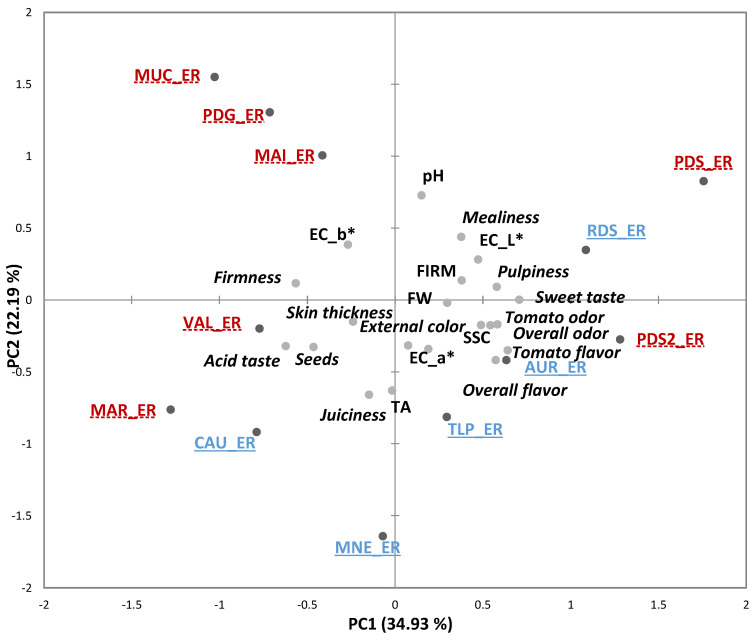
Principal component analysis (PCA) Bi-plot of the sensory and physicochemical parameters of the 12 tomato genotypes grown in Italy and evaluated at the early ripe stage (ER). Sensory descriptors are indicated in *italics*. Modern varieties are in blue, underlined with solid lines; Traditional varieties are in red, underlined with dashed lines. FW = fruit weight, FIRM = firmness (instrumental), SSC = soluble solids content, TA = titratable acidity. Product codes are listed in Table 1.

**Figure 4:**
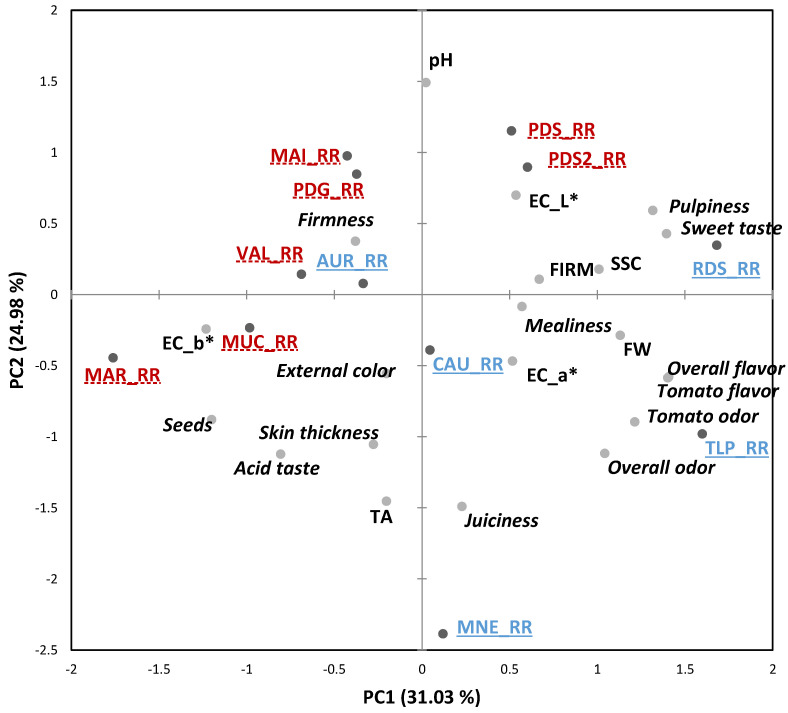
Principal component analysis (PCA) Bi-plot of the sensory and physicochemical parameters of the 12 tomato genotypes grown in Italy and evaluated at red ripe stage (RR). Sensory descriptors are indicated in *italics*. Modern varieties are in blue, underlined with solid lines; Traditional varieties are in red, underlined with dashed lines. FW = fruit weight, FIRM = firmness, SSC = soluble solids content, TA = titratable acidity. Product codes are listed in Table 1.

An overview of the samples’ distribution and relations among sensory and physicochemical variables obtained by PCA is reported in Figure 3 and Figure 4, for varieties evaluated at the ER and RR stages, respectively. At the ER stage, the first two PCs (accounting for 57.11% of the variability) allowed separating the group of varieties (traditional and modern) *PDS*, *PDS2*, *RDS*, *AUR* and *TLP* on the positive side of PC1, from the landraces *MUC*, *PDG*, *MAI*, *VAL*, *MAR*, and the modern variety *CAU*, positioned on the negative semi-axis of PC1 (Figure 3). The variables that most contributed to the variance explained by this axis were “pulpiness”, “overall odor”, “tomato odor”, “sweet taste”, “overall flavor”, “tomato flavor” and SSC, on the positive side, and “seeds”, “acid taste”, “firmness”, on the negative side. The PC2 was mostly related to “juiciness”, TA, and pH, which distinguished mainly *MNE* from *MUC*.

At the RR stage, the correlation bi-plot of the first two PCs (explaining about 56.02% of the variation) showed a similar distribution of the varieties such as the one found at the ER stage, with *RDS*, *PDS* and *PDS2* characterized by higher “sweet taste”, “pulpiness” and SSC, while *TLP* by higher “odor/flavor” notes (Figure 4).

#### 3.3.2. Consumer Data

For the 12 varieties tasted in Italy at the ER stage the OL scores ranged from 4.3 to 5.4/9, and a statistically highly significant genotypic effect was observed (Table 5). However, no special trend of liking due to the varietal group (Traditional/Modern) was found. Tukey’s test revealed the lowest mean OL score for *VAL*, which was significantly different from *PDS* and *CAU*. By contrast, for the eight varieties tested at the RR stage, the OL scores ranged from 5.0 to 5.6/9, and ANOVA showed that neither varietal group nor genotype had a significant effect on OL.

Frequencies of mentioning for the 24 CATA terms are reported in Table S11 (ER stage) and Table S12 (RR stage). The data show that most of the terms (i.e., 21 and 19 terms for the ER and RR products, respectively) had a mean occurrence > 20%. However, Cochran’s Q test revealed that four (ER stage) and 12 (RR stage) terms were not discriminating among samples. At the ER stage, Correspondence Analysis (CA) restituted 67.68% of the inertia (Figure S4). The first axis distinguished the tomato varieties for desirable versus not desirable attributes from the negative semi-axis (“beautiful color”, “I like the appearance”, “sweet”, “juicy”, “aromatic”) to the positive semi-axis (“too much jelly and seeds”, “too much acid”, “unpleasant aftertaste”, “too firm”, “tasteless”). The second axis was mainly drawn by the terms “too big” and “too small”. On this map, a group of modern varieties, *AUR*, *CAU* and *MNE* were perceived by consumers to have the best sensory characteristics including “beautiful color”, “I like the appearance”, “sweet”, “juicy”, “aromatic”. At the RR stage, the Cochran’s Q test revealed that several key terms were not discriminating, making it difficult to get an interpretable map of samples differences (Table S12 and Figure S5).

At both ripening stages, penalty-lift analysis confirmed the main positive and negative drivers of liking identified also in France and Spain, although a higher number of significant negative associations with OL were found. More specifically, at the ER stage, penalty-lift analysis identified “I like its appearance” (2.036), “I like its fleshy aspect” (1.998), “beautiful color” (1.778), “pleasant aroma” (1.746), “sweet”(1.550), “juicy” (1.445) and “strong aroma” (1.429) as potential higher positive directions for OL improvement and “unpleasant aftertaste” (−1.799), “too much acid” (−1.495), “tasteless” (−1.484), “too pale color” (−1.429), “too firm” (−1.146), “too green” (−1.137), “mealy” (−0.960), “dislike appearance” (−0.948), “too soft” (−0.873), “too thick skin” (−0.869), “too much jelly and seeds” (−0.715), “not enough sweet” (−0.674), “too many seeds” (−0.571), “too big” (−0.526), as the main negative attributes.

MFA analysis revealed the relations between DA and CATA configurations at the ER (Figure S2c) and RR stages (Figure S2d), with RV coefficients of 0.60 and 0.69, respectively. For the products tested at the ER stage, among the comparable descriptors and CATA terms, good correlations were found for appearance (seeds), taste (sweet and acid) and texture (mealiness), with correlation coefficients ranging from a minimum of r = 0.63 for “mealiness” to a maximum of r = 0.82 for “sweetness” and “acidity” (data not shown). On the other hand, for the products tested at the RR stage, only the DA descriptors “seeds” and “sweet taste” showed a significant correlation with the correspondent CATA terms “too many seeds” (r = 0.73) and “sweet tomato” (r = 0.87) (data not shown).

## 4. Discussion

### 4.1. Comparison between Traditional and Modern Varieties

Consumer dissatisfaction with the flavor quality of most modern fresh market tomato varieties has promoted the demand for “heirloom” and local or traditional tomato varieties (landraces), which are generally associated with better flavor (“flavor of the past”) and local production [14,19,26]. The Mediterranean region, a secondary center of diversification for tomato, represents one of the regions with the greatest diversity in local tomato varieties. In Europe, some of these landraces are widely recognized, as high quality varieties and can be found in markets throughout the continent [19]. However, in some cases, the landraces have been replaced by commercial varieties (selections or F1 hybrids) that morphologically resemble the correspondent traditional varieties and have the same market destination.

In order to better understand how traditional varieties differ from their modern commercial counterparts, also in terms of consumer preferences and perception, we have conducted a sensory study on 17 European traditional and modern fresh market tomato varieties belonging to different typologies. Different partially overlapping sets of genotypes were grown and evaluated in France, Spain and Italy, as the selection focused on varieties known by consumers of each area. Fruit sensory quality was assessed at three levels: physicochemical, descriptive sensory analyses by trained panels, and consumer tests. Moreover, the influence of the varietal group (Traditional/Modern) on sensory quality and consumer preferences has also been evaluated considering fruits at different ripening stages in Italy, where traditionally tomatoes for fresh consumption are also consumed at the stage of not full ripening.

Our results revealed a significant effect of varietal group on some of the sensory descriptors and physicochemical parameters in all three countries, and, in spite of partially different sets of traits showing a statistically significant effect in each country, a common trend could be observed. Altogether, modern varieties showed higher TA and SSC values (except for SSC in Italy at the ER stage) and were perceived with more intense “overall” and “tomato” flavors (except for “tomato flavor” in Italy at the RR stage) than the traditional ones. In France, firmness (instrumental and sensory) was significantly higher in the Modern group than in the Traditional one, a result in line with earlier studies [7], and with firmness being a key trait in fresh market tomato breeding.

Several studies have compared sensory properties and/or sensory-related instrumental traits of traditional and modern tomato varieties/breeding lines, focusing on specific types of landraces from Southern Italy [20,24], Eastern Spain (*Muchamiel* and *De la Pera*) [23,43,44,45], or the Andes [25,26]. In some cases, the traditional varieties showed higher fruit sensorial qualities than the modern controls along with significantly higher contents of most or some of the aroma volatiles studied [25,26,44]. However, it is worth noting that the intra-landrace genetic variation for sensory traits as well as the choice of the modern controls might bias the conclusions of the comparisons, as might have been the use of a processing tomato cultivar in comparison with good-tasting Andean landraces [26], or of modern counterparts not belonging to the specific typologies of the analyzed landraces [24,45]. Nevertheless, even when the specific modern counterparts are used, the traditional varieties do not always show significantly better sensory characteristics [20].

In our study, consumer tests have also been conducted on a subset of the varieties, and, consistent with the sensory and physicochemical profiles, in France and Spain, significantly higher OL was found for the modern variety group with respect to the traditional one. However, in France, the overall significant varietal group effect on OL was mainly due to the “Oxheart” and “Marmande” typologies, as no significant differences were observed for the “Round” typology. In Spain, the significant varietal group effect on OL was due to all three typologies tested (“Oxheart”, “Oxheart Liguria” and “Marmande”), although OL of *PDS*, one of the “Oxheart” traditional varieties, was not significantly different from that of *CAU*, one of the “Oxheart” modern counterparts used for this typology. In Italy, instead, the varietal group effect on OL was not significant at both ripening stages.

Overall, our data indicate that, at least within most of the tomato fresh market typologies analyzed in this study, modern breeding for consumer-driven quality traits is leading to satisfactory results. Furthermore, considering that some of the preferred modern varieties (i.e., *AUR*, *CAU* and *MNE*) are F1s carrying several disease resistance genes (DRGs), these results also provide a good indication that it is possible to maintain fruit sensory quality while increasing resilience, as also shown by Alonso et al. [23].

Our findings are also in line with those by Schouten et al. [18] who studied a collection of 90 tomato varieties that were commercially released between 1950 and 2016 in NW Europe. Although not based on sensory evaluations, their data indicate that starting from the beginning of 1990s, tomato breeding in NW Europe has led to a second boost of diversity, specifically in fruit types and improved flavor composition. In contrast, [14] comparing a large set of modern commercial varieties to old, heirloom tomatoes, concluded that the modern varieties had a lower flavor quality compared to the old varieties due to breeding, although they had also found that heirlooms are not always characterized by great taste [27].

### 4.2. Effects of Different Growing Conditions

Several studies have explored the effect of different environmental/growing conditions on sensory and flavor-related instrumental measures of tomato quality [7,28,29,30,46,47,48,49]. Some of these past studies have demonstrated that tomato fruit quality is highly affected by environmental conditions and that most of the quality traits are highly polygenic and show low heritability [7,49]. Nevertheless, the results depend on numerous factors including the set of genotypes, traits and sensory descriptors analyzed, the different environments and growing systems tested, the maturity stage, and the time of harvest, just to list a few. In our study, the effects of different GCs on tomato fruit sensory quality were assessed in France (S/SH) and in Spain (GH/OF).

Considering average differences between growing conditions, a significant GC effect was observed for several physicochemical and sensory traits in both countries. However, the number of common objective parameters and sensory descriptors significantly affected by the GCs was higher in France (100.0% and 77.8%, respectively) than in Spain (33.3% and 44.4%, respectively). This might be partly caused by the more diverse cultivation systems adopted in France, which included also different plant nutrition regimes. In spite of the significant GC effect observed on many of the measured sensory and instrumental variables, the genotypic effect was significant for all the measured variables in both countries.

On average, in both countries, four of the common sensory descriptors (“overall odor”, “acid taste”, “firmness” and “mealiness”) and two of the common physicochemical parameters (FIRM and SSC) were significantly affected by GCs, while no significant GC effect was observed on “sweet taste” and “skin thickness”. It is worth noting that GCs had a significant effect on SSC in both countries, and also on all the other sweet-related traits measured in FR (sucrose, glucose and fructose), while no significant effect was observed on perceived “sweetness”. The lack of strong correspondence between instrumental and sensory data is not unusual and can be partly due to complex synergistic and antagonistic interaction dynamics of sugars, acids, and volatile chemicals with human receptors, that can change the level of detection of individual components, especially in complex foods like tomatoes [14].

Considering all the sensory descriptors analyzed, in both countries, a similar proportion of flavor-related and texture-related traits were significantly affected by GCs.

Instead, a previous study by Carli et al. [28] found more pronounced GC effects on taste-related traits (saltiness, sourness, sweetness), than on texture-related traits (juiciness, granularity, hardness). Similarly, Casals et al. [30], who also tested open field vs. greenhouse conditions, reported a more pronounced environmental effect on taste-related traits (“sweetness”, “acidity”, “taste intensity”), and no significant effect on texture-related traits (“mealiness”, “firmness” and “skin persistence”).

In addition, a significant G × GC interaction was observed for most (France) and many (Spain) of the sensory and physicochemical parameters analyzed, indicating that the GC effect was not consistent across genotypes. Significant G × E interactions of tomato quality traits have been reported in several earlier studies [7,28,30,47,49], and in keeping with some of these reports, the G × GC interactions observed in the present study were usually less significant than the main genotypic or environmental effects [7,49].

In both countries, the PCA plots did not reveal any clear separation of the products according to the different growing systems, reinforcing the results obtained from the ANOVAs, in the sense that though the GCs had an effect on both sensory and physicochemical data, overall the GC effect was lower than the genotypic effect, and there was a significant G × GC effect for most variables.

The effect of the different GCs on consumer OL was also explored in our study. The results obtained in France and Spain highlight that overall there was no GC effect on consumer OL, which was genotype-oriented in both countries. In line with our results, a study conducted on organically and conventionally grown tomatoes did not show significant differences in consumer liking [50]. In contrast, Causse et al. [7] observed a significant effect of growing conditions (open field vs. greenhouse) on consumer preferences of fresh market tomato hybrids, with a less important difference reported for the small-fruited tomatoes compared to the large-fruited ones. In addition, Gilsenan et al. [51] reported that the consumer panel was able to distinguish a perceptible difference between organically and conventionally farmed tomatoes, preferring the taste of the conventional cherry tomatoes for their higher sugar content and sweetness.

### 4.3. Comparison of Product Configurations and Drivers of Liking Based on CATA

In the last decade, the use of novel consumer-based sensory characterization methodologies has become a valuable alternative tool to traditional DA, with CATA questions being one of the most popular of these methodologies [32]. In this study, we aimed to obtain consumer-based sensory profiles of European traditional and modern fresh market tomato varieties belonging to different typologies using the CATA method, and to compare these product configurations with those obtained by DA with trained assessors. Although the CATA questions method has already been used for sensory characterization of cherry tomatoes with consumers and chefs [52], to the best of our knowledge, this is the first study in which the two sensory methodologies, DA by trained panels and CATA questions by consumers, are compared on fresh market tomatoes.

Our results show that in France and Spain, all the CATA terms were able to detect differences in consumer perception of the sensory characteristics of the evaluated tomato products. In Italy, consumers found more statistically significant differences at the ER stage (83% of the terms) than the RR stage (50% of the terms); these results could in part be due to the lower sensory space provided by the eight RR products compared to the 12 ER products. The majority of the most cited terms were common to the three countries, although in Italy the frequency of citation of the terms was generally higher. Overall, these results confirm the CATA method as a powerful sensory profiling tool to apprise consumer perceptions of differences in the sensory attributes among the studied tomato products.

Results from DA with trained panels and CATA questions with consumers were compared in each country. The RV coefficients between product configurations in the first two dimensions of the MFA were always significant, with values ranging from 0.60 (Italy, ER stage) to 0.78 (France). These results show agreement between the two methods, even though the CATA terms were not identical to those used by the trained panel. Overall, our results are in line with previous studies, conducted on products with different sensory complexities, which have shown the two methods to generate comparable product configurations [33,35,36,53]. The lower agreement between DA and CATA configurations observed in Spain and Italy might be attributed to smaller differences within the product sets evaluated in both countries compared to those assessed in France. These results confirm other authors’ findings of a better agreement between CATA and DA configurations when differences between samples are large [33,34,35,36,53].

In this study, penalty-lift analysis was conducted on CATA questions to identify drivers of consumer liking. The analyses have allowed the identification of drivers of liking/disliking common to at least two countries. Specifically, ”aromatic”, ”sweetness”, “fleshy aspect”, “good appearance”, “beautiful color” and “juiciness” were the main common positive drivers, while “bland taste”, “too thick skin” and “not enough sweet” the main negative ones, with somewhat different order of importance between the three countries. In France and Italy, the attribute with the strongest positive impact on OL was “aromatic tomato”, while in Spain it was “sweet tomato”. On the other hand, for all three countries, the attribute with the strongest negative impact on OL was “bland/no taste”.

These results are in line with previous studies, based on sensory maps provided by classic DA with trained panels, that have also highlighted the importance of color and appearance to consumer liking of fresh tomatoes [8,54,55], as well as of sweetness [52,54,55], flavor [7,8] and juiciness [9,54].

However, it is worth highlighting that specific clusters of consumers may have specific preferences, as has been reported in several studies conducted on fresh market tomatoes [8,9,52,54,55], and that the drivers of liking are also influenced by the type of fruit tested [7].

## 5. Conclusions

Results from the present work indicate that the genotypic effect on sensory and physicochemical profiles was generally stronger than the growing condition (GC) effect. Consistent with these results, overall, a strong genotypic effect on consumer OL was found, while the GC effect on OL was not significant. Altogether, in France and Spain acceptance of modern varieties was higher than for the traditional ones. These results indicate that, at least for most of the varietal typologies analyzed in this study, modern breeding for tomato sensory quality is in the right direction.

Moreover, this study improves our comprehension of the relationship between consumers’ perceptions and preferences and confirms the CATA method with consumers as a powerful sensory profiling tool to apprise consumer perceptions of differences in the sensory attributes among the studied tomato products. Finally, penalty-lift analysis based on CATA questions allowed the identification of positive and negative drivers of consumer liking that provide useful leads for the development of fresh market tomato varieties that satisfy European consumers’ expectations.

## Figures and Tables

**Table 1:** List of fresh market tomato varieties (Traditional and Modern counterparts) analyzed by trained and consumer panels in each country and in different growing conditions or ripening stages.

Variety ^a^	Traditom Pedigree N. ^b^	Code	Varietal Group ^c^	Origin/ Seed Company	Varietal Typology ^d^	Tested in France ^e^		Tested in Italy ^f^		Tested in Spain ^g^	
						S	HS	ER	RR	GH	OF
Maillane	TRMO0450	*MAI*	T	France	Oxheart	X *	X *	X *	X *	X *	X *
Valenciana	TRVA0020	*VAL*	T	Spain	Oxheart	X *	X	X *	X *	X *	X *
Pomodoro di Sorrento	TRPO0190	*PDS*	T	Italy	Oxheart	X	X	X *	X *	X *	X
Pomodoro di Sorrento (L2)	NA	*PDS2*	T	Italy	Oxheart			X			
Pera de Girona	TRCA0010	*PDG*	T	Spain	Oxheart (Lig.)	X	X	X *	X *	X *	X
Marmande	TRMO0010	*MAR*	T	France	Marmande	X *	X *	X *	X *	X *	X *
Muchamiel	TRVA0010	*MUC*	T	Spain	Marmande			X *	X	X *	X
St. Pierre	TRMO0020	*STP*	T	France	Round	X *	X *				
Cauralina (F1)	TRMC0060	*CAU*	M	Gautier Semences	Oxheart	X *	X *	X *	X *	X *	X *
Toi Ls Pan (F1)	TRMC0700	*TLP*	M	Lamboseed	Oxheart			X *	X	X	X
Rosa di Sorrento (C.S.)	TRMC0710	*RDS*	M	Semencoop	Oxheart			X *	X		
Aurea (F1)	TRMC0730	*AUR*	M	De Ruiter	Oxheart (Lig.)	X	X	X *	X *	X *	X
Marbonne (F1)	TRMC0720	*MNE*	M	Gautier Semences	Marmande	X *	X *	X *	X *	X *	X *
Marmalindo (F1)	TRMC0520	*MLD*	M	Nunhems	Marmande					X *	X *
Garance (F1)	TRMC0450	*GAR*	M	INRA	Round	X *	X *				
Climberley (F1)	TRMC0760	*CLI*	M	Syngenta	Round	X	X *				
Hybride INRA (F1)	TRMC0490	*HYB*	M	INRA	Round	X	X				

^a^ L2 = Landrace 2; C.S. = Commercial Selection. ^b^ NA = not available. ^c^ T = Traditional; M = Modern. ^d^ Lig. = Liguria. ^e^ S: soil; HS: soilless. ^f^ ER: early ripe stage; RR: red ripe stage. ^g^ GH: greenhouse; OF: open field. * indicates products also used for consumer tests.

**Table 2:** Sensory descriptors determined by the trained taste panel and list of CATA terms in the consumer tests in each country.

Descriptive Analysis Terms and Definitions		FR	ES	IT	CATA Terms	FR	ES	IT
**Visual examination**								
External color	Proportion of external green-red color of the tomato			X	Beautiful color	X	X	X
Color intensity	Visual examination of the tomato placed on the peduncular side. For tomatoes with another color than red (green, orange, yellow, pink, streaked …), do not score this criterion	X			I like its appearance	X	X	X
Seeds	Amount of seeds in the internal locular portion			X	Too big	X	X	X
Pulpiness	Visual examination of thickness of the mesocarp and radial septa in relation to the seminal loggias of endocarp			X	I like its fleshy aspect	X	X	X
Size	Visual examination of the diameter of the tomato placed blossom end up	X			I do not like its appearance	X	X	X
Rib aspect	Visual examination of ribs extent (tomato turned over, with stem scar visible)	X			Hollow fruit	X	X	X
					Too small	X	X	X
					Too much jelly and seeds	X	X	X
					Too green			X
					Too red			X
					Too pale color			X
**Texture examination**								
Firmness	Force needed to penetrate tomato flesh with the teeth	X	X	X	Juicy	X	X	X
Firmness to cut	Resistance to penetrate tomato flesh with a knife	X			Too thick Skin	X	X	X
Juiciness	Amount of juice release by the piece of tomato during chewing	X	X	X	Too firm	X	X	X
Mealiness	Describes the feeling mealy tomato perceived during chewing	X	X	X	Mealy	X	X	X
Skin thickness	Thickness of the tomato skin perceived during chewing	X	X	X	Too soft	X	X	X
Crunchiness	Force needed when applied to the tomato to create a crunchy sound	X			Too many seeds	X	X	X
Meltiness	Ease with which the piece of tomato melts in the mouth between the tongue and the palate	X						
**Flavor examination**								
Overall odor	Overall impression perceived by receptors of the olfactory system (via orthonasal) after cutting	X	X	X	Sweet		X	X
Tomato odor	Odor associated with the aromatic compounds characterizing ripe tomato			X	Too much acid	X	X	X
Sweet taste	Basic taste produced by the amount of sugar (e.g., fructose or glucose)	X	X	X	Not enough sweet	X	X	X
Acid taste	Basic taste produced by the amount of organic acids (e.g., citric or malic acids)	X	X	X	Bland/no taste	X	X	X
Overall flavor	Overall impression originated by the volatiles released during chewing and perceived retro-nasally	X	X	X	Not pleasant aftertaste	X	X	X
Tomato flavor	Flavor sensation associated with the aromatic compounds characterizing ripe tomato	X	X	X	Pleasant aroma	X		X
					Aromatic tomato/strong aroma	X	X	X

**Table 3:** Analysis of variance and mean overall liking (OL) scores (*n* = 100) of tomato samples harvested at red ripe stage in France (9-point hedonic scale).

Genotype	Varietal Group	Varietal Typology		OL		Genotype (G)	Varietal Group (V)		Growing Conditions (GC)		(G) × (GC)	(V) × (GC)
			Overall Mean	S (S.E.)	HS (S.E.)	F ^(1)^	F ^(1)^	OL	F ^(1)^	OL	F ^(1)^	F ^(1)^
MNE	M	Marmande	6.6 ^a^	6.6 (0.14)	6.5 (0.17)	9.16 ***	20.17 ***	M = 6.3 ^a^	0.53	S = 6.1	2.98 *	0.95
STP	T	Round	6.3 ^ab^	6.3 (0.18)	6.4 (0.18)			T = 5.8 ^b^		HS = 6.0		
CAU	M	Oxheart	6.2 ^ab^	6.3 (0.19)	6.1 (0.18)							
GAR	M	Round	6.1 ^ab^	5.8 (0.19)	6.5 (0.17)							
CLI	M	Round	6.1 ^ab^	-	6.1 (0.18)							
MAR	T	Marmande	6.0 ^bc^	6.3 (0.17)	5.7 (0.19)							
VAL	T	Oxheart	5.4 ^cd^	5.4 (0.22)	-							
MAI	T	Oxheart	5.3 ^d^	5.5 (0.20)	5.2 (0.20)							

Marbonne (MNE), St Pierre (STP), Cauralina (CAU), Garance (GAR), Climberley (CLI), Marmande (MAR), Valenciana (VAL), Maillane (MAI); M = Modern variety; T = Traditional variety; S = soil; HS = soilless. ^(1)^ F value; * *p* ≤ 0.05; *** *p* ≤ 0.001. Within each testing condition, overall liking means with common superscript letters are not significantly different (*p* ≤ 0.05) according to the Tukey’s (HSD) post hoc test.

**Table 4:** Analysis of variance and mean overall liking scores (*n* = 123) of tomato samples harvested at the red ripe stage in Spain (10-point hedonic scale).

Genotype	Varietal Group	Varietal Typology		OL		Genotype (G)	Varietal Group (V)		Growing Conditions (GC)		(V) × (GC)	(G) × (GC)
			Overall Mean	GH (S.E.)	OF (S.E.)	F ^(1)^	F ^(1)^	OL	F ^(1)^	OL	F ^(1)^	F ^(1)^
PDS	T	Oxheart	6.7 ^a^	6.7 (0.15)	^-^	22.91 ***	83.60 ***	M = 6.6 ^a^	0.43	G = 6.1	3.56 ***	0.68
MLD	M	Marmande	6.7 ^a^	6.6 (0.15)	6.7 (0.15)			T = 5.8 ^b^		OA = 6.0		
AUR	M	Oxheart (Lig.)	6.5 ^a^	6.5 (0.18)	-							
CAU	M	Oxheart	6.5 ^a^	6.8 (0.16)	6.1 (0.17)							
MNE	M	Marmande	6.3 ^a^	6.2 (0.16)	6.4 (0.16)							
VAL	T	Oxheart	5.8 ^b^	5.9 (0.17)	5.8 (0.17)							
MAR	T	Marmande	5.7 ^bc^	5.5 (0.16)	5.8 (0.18)							
MAI	T	Oxheart	5.7 ^bc^	6.0 (0.16)	5.4 (0.17)							
PDG	T	Oxheart (Lig.)	5.4 ^c^	5.4 (0.20)	-							
MUC	T	Marmande	4.4 ^d^	4.4 (0.19)	-							

Pomodoro di Sorrento (PDS), Marmalindo (MLD), Aurea (AUR), Cauralina (CAU), Marbonne (MNE), Valenciana (VAL), Marmande (MAR), Maillane (MAI), Pera de Girona (PDG), Muchamiel (MUC). Lig. = Liguria; M = Modern variety; T = Traditional variety; GH = greenhouse; OF = open field. ^(1)^ F value; * *p* ≤ 0.05; *** *p* ≤ 0.001. Within each testing condition, overall liking means with common superscript letters are not significantly different (*p* ≤ 0.05) according to the Tukey’s (HSD) post hoc test.

**Table 5:** Analysis of variance and mean overall liking (OL) (*n* = 107) scores of tomato samples harvested at the early ripe (ER) and red ripe (RR) stages in Italy (9-point hedonic scale).

ER Stage							RR Stage						
Genotype (G)	Varietal Group	Varietal Typology	OL (S.E.)	G	Varietal Group		Genotype (G)	Varietal Group	Varietal Typology	OL (S.E.)	G	Varietal Group	
				F ^(1)^	F ^(1)^	OL					F ^(1)^	F ^(1)^	OL
AUR	M	Oxheart(Lig.)	4.9 ^ab^ (0.20)	2.33 **	2.54	M = 5.1	AUR	M	Oxheart (Lig.)	5.3 (0.17)	0.97	0.11	M = 5.4
CAU	M	Oxheart	5.3 ^a^ (0.20)			T = 4.9	CAU	M	Oxheart	5.6 (0.18)			T = 5.3
MAI	T	Oxheart	4.9 ^ab^ (0.20)				MAI	T	Oxheart	5.0 (0.18)			
MNE	M	Marmande	5.2 ^ab^ (0.19)				MNE	M	Marmande	5.2 (0.19)			
MAR	T	Marmande	5.3 ^ab^ (0.19)				MAR	T	Marmande	5.4 (0.17)			
MUC	T	Marmande	4.6 ^ab^ (0.20)				-	-	-	-			
PDG	T	Oxheart(Lig.)	4.9 ^ab^ (0.20)				PDG	T	Oxheart (Lig.)	5.3 (0.18)			
PDS2	T	Oxheart	4.9 ^ab^ (0.19)				-	-	-	-			
PDS	T	Oxheart	5.4 ^a^ (0.19)				PDS	T	Oxheart	5.4 (0.17)			
RDS	M	Oxheart	5.1 ^ab^ (0.21)				-	-	-	-			
TLP	M	Oxheart	4.9 ^ab^ (0.21)				-	-	-	-			
VAL	T	Oxheart	4.3 ^b^ (0.21)				VAL	T	Oxheart	5.5 (0.19)			

Aurea (AUR), Cauralina (CAU), Maillane (MAI), Marbonne (MAR), Marmande (MAR), Pera de Girona (PDG), Pomodoro di Sorrento (PDS), Valenciana (VAL). M = Modern variety; T = Traditional variety. Lig. = Liguria. ^(1)^ F-value; ** *p* ≤ 0.01; Within each testing condition, overall liking means with common superscript letters are not significantly different (*p* ≤ 0.05) according to the Tukey’s (HSD) post hoc test.

## Supplementary Materials

The following are available online at https://www.mdpi.com/article/10.3390/foods10112521/s1, Figure S1: Bi-plot resulting from Correspondence Analysis performed on frequency table of consumers’ responses to Check-All-That-Apply (CATA) questions for tomatoes harvested at the red ripe stage in France; Figure S2: Consensus MFA Maps with superimposed points from Descriptive analysis (DA) and CATA questions; Figure S3: Bi-plot resulting from Correspondence analysis performed on frequency table of consumers’ responses to CATA questions for tomatoes harvested at the red ripe stage in Spain; Figure S4: Bi-plot resulting from correspondence analysis performed on frequency table of consumers’ responses to CATA questions for tomatoes harvested at the early ripe stage (ER) in Italy; Figure S5: Bi-plot resulting from correspondence analysis performed on frequency table of consumers’ responses to CATA questions for tomatoes harvested at red ripe (RR) stage in Italy; Table S1: Analysis of variance and means of sensory descriptors for varietal group (Modern/Traditional), growing conditions (Soil/Soilless) and genotypes of tomato samples harvested at the red ripe stage in France (scale from 0 to 10, discontinuous; values are averages of two replicates); Table S2: Analysis of variance and means of physicochemical parameters for varietal group (Modern/Traditional), growing condition (Soil/Soilless) and genotypes of tomato samples harvested at the red ripe stage in France (values are averages of three replicates); Table S3: Contingency table of the frequencies of mentions by French consumers (*n* = 100 eligible consumers) across all 14 tomato samples, evaluated at the red ripe stage, for individual terms of the Check-All-That-Apply (CATA) questions; Table S4: Analysis of variance and means of sensory descriptors for varietal group (Modern/Traditional), growing condition (Greenhouse/Open Field) and genotypes of tomato samples harvested at the red ripe stage in Spain (scale from 0 to 10, semi-structured continuous; values are averages of two replicates); Table S5: Analysis of variance and means of physicochemical parameters for varietal group (Modern/Traditional), growing condition (Greenhouse/Open Field) and genotypes of tomato samples harvested at the red ripe stage in Spain (values are averages of a minimum of two replicates); Table S6: Contingency table of the frequencies of mentions by Spanish consumers (*n* = 115 eligible consumers) across all 16 tomato samples, evaluated at the red ripe stage, for individual terms of the CATA questions; Table S7: Analysis of variance and means of sensory descriptors for varietal group (Modern/Traditional) and genotypes of tomato samples harvested at the early ripe stage (ER) in Italy (scale from 0 to 9, continuous; values are averages of two replicates); Table S8: Analysis of variance and means of physicochemical parameters for varietal group (Modern/Traditional) and genotypes of tomato samples harvested at the early ripe (ER) stage in Italy (values are averages of four replicates); Table S9: Analysis of variance and mean of sensory descriptors for varietal group (Modern/Traditional) and genotypes of tomato samples harvested at the red ripe stage (RR) in Italy (scale from 0 to 9, continuous; values are averages of two replicates; Table S10: Analysis of variance and means of physicochemical parameters for varietal group (Modern/Traditional) and genotypes of tomato samples harvested at the red ripe stage (RR) in Italy (values are averages of four replicates); Table S11: Contingency table of the frequencies of mentions by Italian consumers (*n* = 107 eligible consumers) across all 12 tomato samples, evaluated at the early ripe (ER) stage, for individual terms of the CATA questions; Table S12: Contingency table of the frequencies of mentions by Italian consumers (*n* = 107 eligible consumers) across all 8 tomato samples, evaluated at the red ripe (RR) stage, for individual terms of the CATA questions.
