# Supplementary material for: Sensory Traits and Consumer’s Perceived Quality of Traditional and Modern Fresh Market Tomato Varieties: A Study in Three European Countries
Source: Foods. 2021 Oct 21;10(11):2521. doi: 10.3390/foods10112521 (PMC8620402; doi:10.3390/foods10112521)
Supplement: Supplementary file 1 [file foods-10-02521-s001.zip › foods-1409015-supplementary.pdf]

SUPPLEMENTARY MATERIAL

Sensory traits and consumer's perceived quality of traditional and modern fresh market tomato varieties: a Study in three European countries

Fiorella Sinesio, Maria Cammareri, Valentine Cottet, Lilian Fontanet, Michel Jost, Elisabetta Moneta, Samuela Palombieri, Marina Peparaio, Roser Romero del Castillo, Eleonora Saggia Civitelli, Patrizia Spigno, Antonella Vitiello, Brigitte Navez, Joan Casals, Mathilde Causse, Antonio Granell, Silvana Grandillo

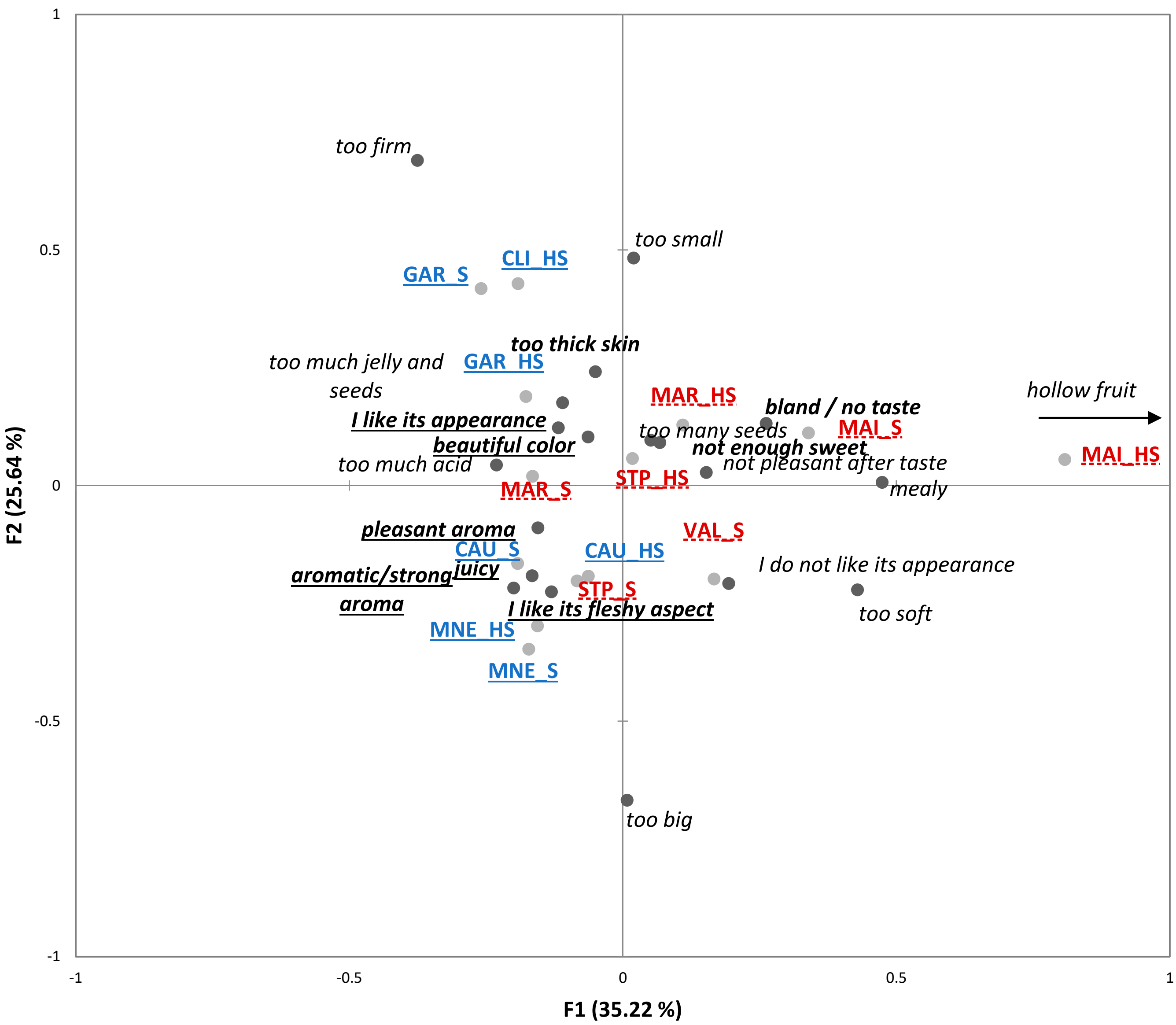

Figure S1. Bi-plot resulting from Correspondence Analysis performed on frequency table of consumers' responses to Check-All-That-Apply (CATA) questions for tomatoes harvested at the red ripe stage in France.

The items that have significant positive association with overall liking from a penalty-lift analysis are reported in bold italics and underlined; negative associations are reported in bold italics. Modern varieties are in blue, underlined with solid lines; Traditional varieties are in red, underlined with dashed lines. S= soil; HS=soilless.

Aurea (AUR), Cauralina (CAU), Climberley (CLI), Garance (GAR), HybInra (HYB) Maillane (MAI), Marbonne (MNE), Marmande (MAR), Pera de Girona (PDG), Pomodoro di Sorrento (PDS), St Pierre (STP), Valenciana (VAL)

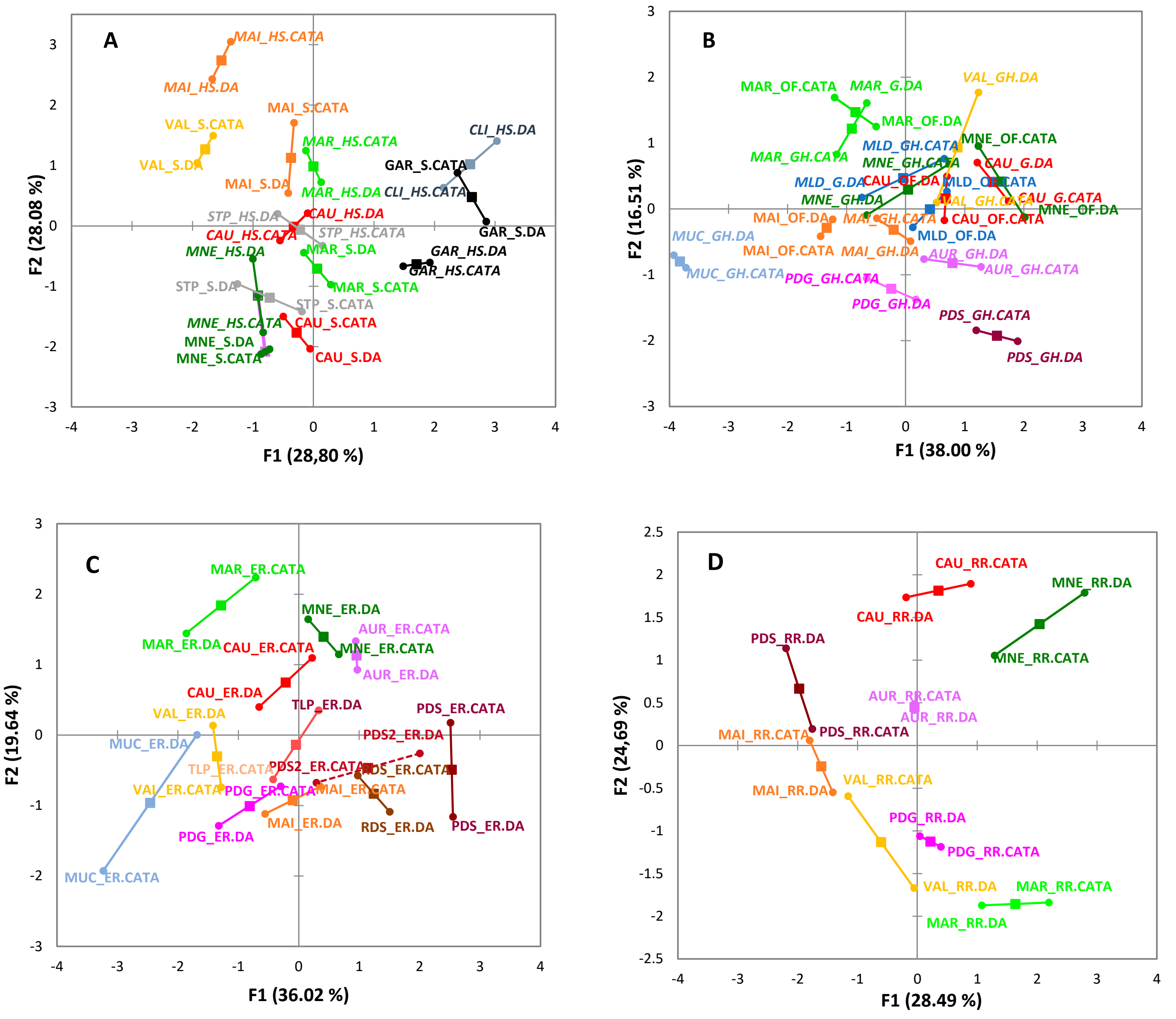

Figure S2. Consensus MFA Maps with superimposed points from Descriptive analysis (DA) and CATA questions.

A: France; B: Spain; C: Italy ER stage; D: Italy RR stage. S= Soil; HS=Soilless. GH=Greenhouse; OF=Open Field. Product codes are listed in Table 1.

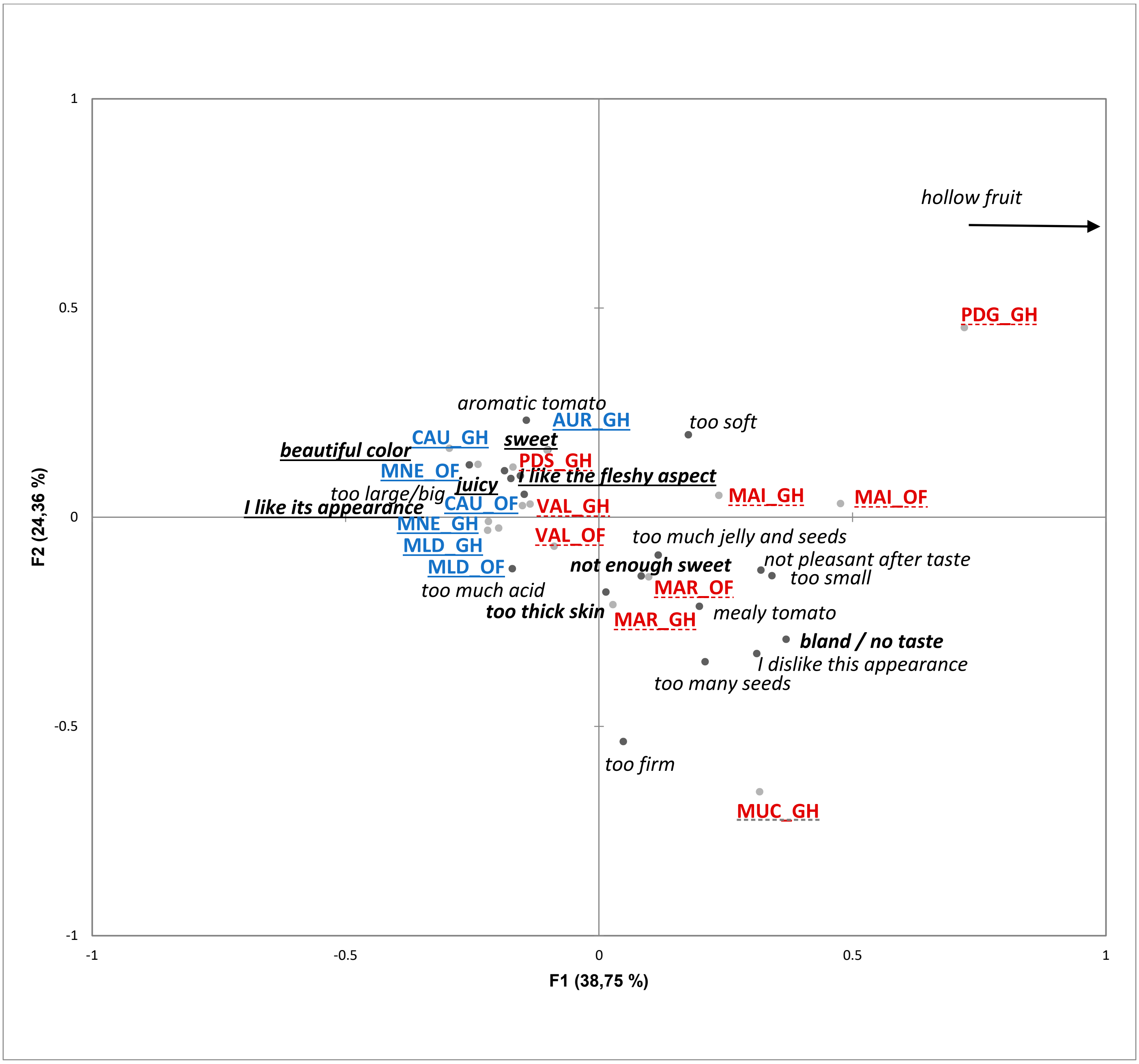

Figure S3. Bi-plot resulting from Correspondence analysis performed on frequency table of consumers' responses to CATA questions for tomatoes harvested at the red ripe stage in Spain.

The items that have significant positive association with overall liking from a penalty-lift analysis are reported in bold italics and underlined; negative associations are reported in bold italics. Modern varieties are in blue, underlined with solid lines; Traditional varieties are in red, underlined with dashed lines. GH=greenhouse; OF=open field.

Aurea (AUR), Cauralina (CAU), Maillane (MAI), Marmande (MAR), Marmalindo (MLD), Marbonne (MNE), Muchamiel (MUC), Pera de Girona (PDG), Pomodoro di Sorrento (PDS), Valenciana (VAL).

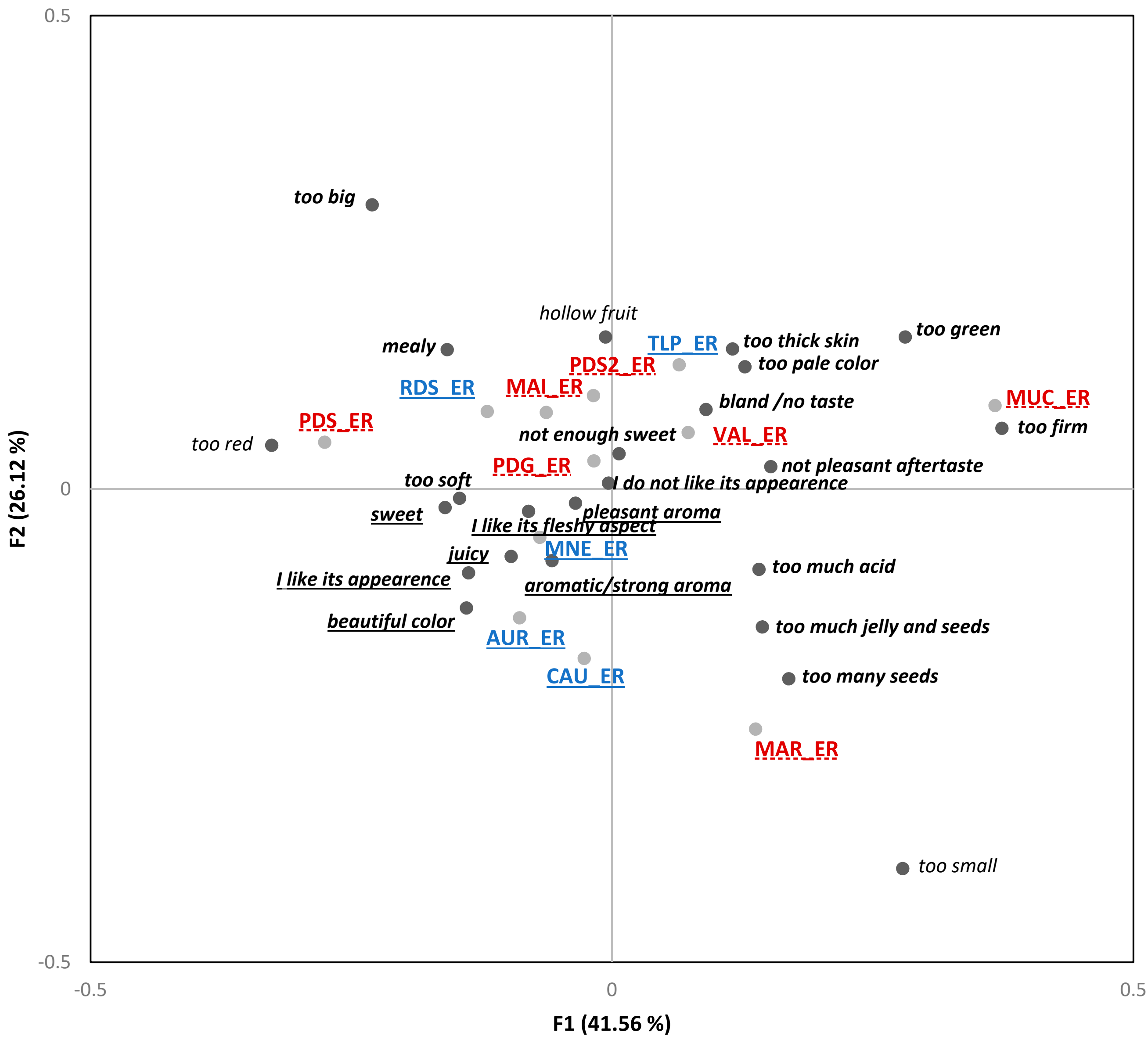

Figure S4. Bi-plot resulting from correspondence analysis performed on frequency table of consumers' responses to CATA questions for tomatoes harvested at the early ripe stage (ER) in Italy.

The items that have significant positive association with overall liking from a penalty-lift analysis are reported in bold italics and underlined; negative associations are reported in bold italics. Modern varieties are in blue, underlined with solid lines; Traditional varieties are in red, underlined with dashed lines.

Aurea (AUR), Cauralina (CAU), Maillane (MAI), Marmande (MAR), Marbonne (MNE), Muchamiel (MUC), Pera de Girona (PDG), Pomodoro di Sorrento (PDS), Pomodoro di Sorrento (Landrace 2) (PDS2), Rosa di Sorrento (RDS), Toi Ls Pan (TLP), Valenciana (VAL).

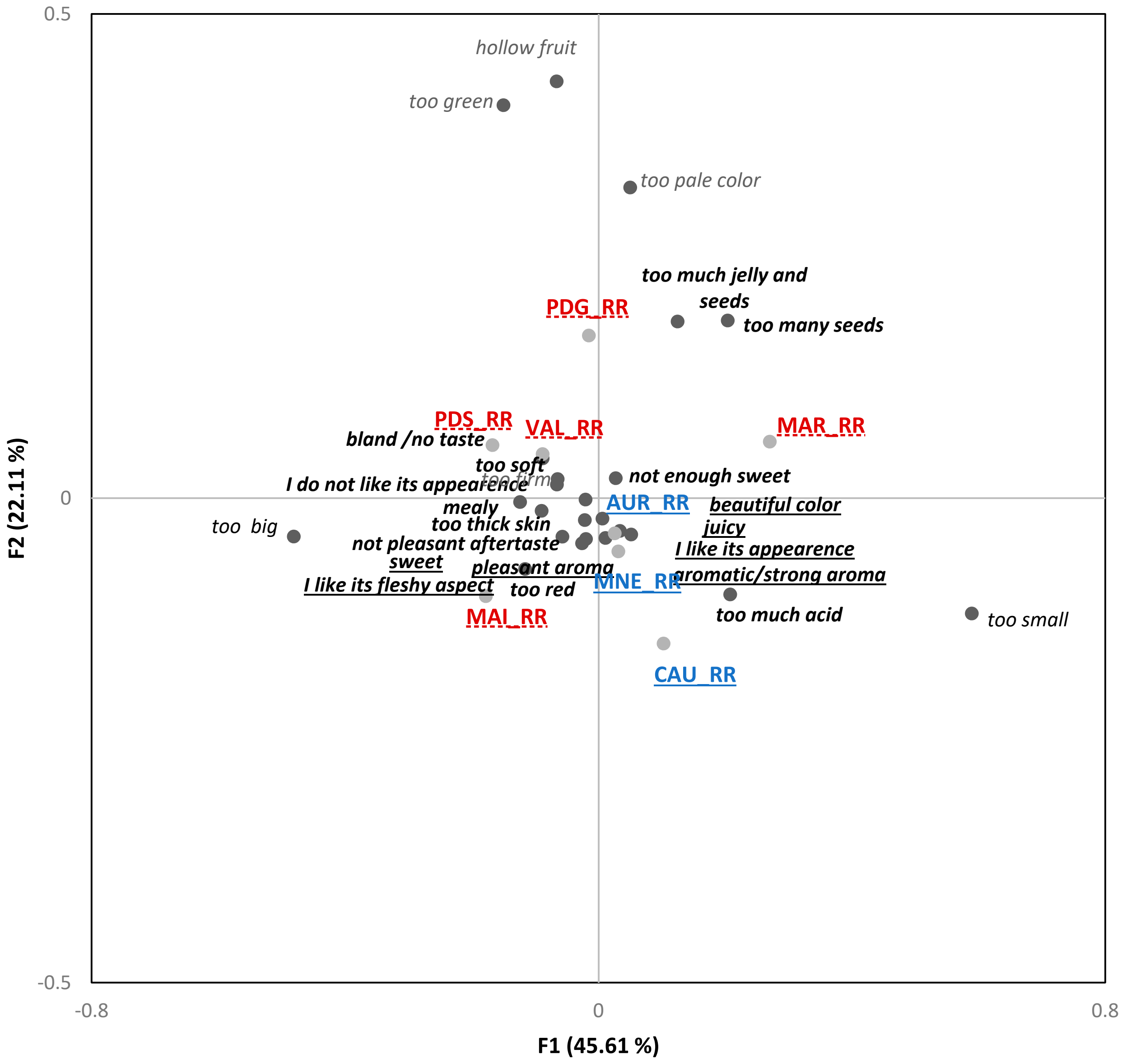

Figure S5. Bi-plot resulting from correspondence analysis performed on frequency table of consumers' responses to CATA questions for tomatoes harvested at red ripe (RR) stage in Italy.

The items that have significant positive association with overall liking from a penalty-lift analysis are reported in bold italics and underlined. Negative associations are reported in bold italics. Modern varieties are in blue, underlined with solid lines; Traditional varieties are in red, underlined with dashed lines.

Aurea (AUR), Cauralina (CAU), Maillane (MAI), Marmande (MAR), Marbonne (MNE), Muchamiel (MUC), Pera de Girona (PDG), Pomodoro di Sorrento (PDS), Pomodoro di Sorrento (Landrace 2) (PDS2), Rosa di Sorrento (RDS), Toi Ls Pan (TLP), Valenciana (VAL).

Table S1. Analysis of variance and means of sensory descriptors for varietal group (Modern/Traditional), growing conditions (Soil/Soilless) and genotypes of tomato samples harvested at the red ripe stage in France (scale from 0 to 10, discontinuous; values are averages of two replicates).

Sensory descriptor	Varietal group (V) $^{(1)}$	Varietal group (V) M	Varietal group (V) T	Growing Condition (GC) $^{(1)}$	Growing Condition (GC) S	Growing Condition (GC) HS	(V)*(GC) $^{(1)}$	Genotype (G) $^{(1)}$	(G)*(GC) $^{(1)}$	Oxheart (Liguria) **AUR**	Oxheart (Liguria) PDG	Oxheart **CAU**	Oxheart MAI	Oxheart PDS	Oxheart VAL	Marmande **MNE**	Marmande MAR	Round **CLI**	Round **GAR**	Round **HYB**	Round STP
Color intensity	89.89***	6.3^{a}	4.5^{b}	2.07	5.5	5.3	0.64	60.87***	2.18*	5.9^{bc}	5.4^{cd}	5.9^{bc}	5.3^{cd}	0.1^{e}	4.4^{d}	5.9^{bc}	5.9^{bc}	6.0^{bc}	6.9^{ab}	7.4 ^{a}	6.1^{bc}
Size	9.58**	6.3^{b}	6.8^{a}	4.58*	6.4^{b}	6.7^{a}	0.23	39.23***	1.95*	7.3^{a}	7.5^{a}	7.5^{a}	6.2^{b}	7.7^{a}	7.5^{a}	8.1^{a}	5.5^{bc}	4.7 ^{cd}	4.5 ^{d}	5.7^{b}	6.1^{b}
Rib aspect	0.63	3.7	3.5	1.43	3.5	3.7	1.32	58.60***	0.82	7.1^{a}	5.6^{b}	4.1^{c}	3.3^{cde}	2.6^{de}	3.7^{cd}	5.8^{b}	3.6^{cd}	0.9 ^{g}	1.4^{fg}	2.6^{de}	2.2^{ef}
Firmness to the cut	108.83***	6.1^{a}	4.6^{b}	19.53***	5.1^{b}	5.7^{a}	0.97	21.17***	2.79**	5.3^{cd}	4.7^{de}	6.0^{bc}	4.0^{e}	4.6^{de}	5.4^{cd}	5.3^{cd}	5.3^{cd}	7.3^{a}	6.6 ^{ab}	6.3^{abc}	3.7^{e}
Overall odor	7.20**	4.9^{a}	4.5^{b}	8.51**	4.9^{a}	4.5^{b}	0.97	4.60***	0.76	4.3^{bc}	4.4^{abc}	5.0^{ab}	5.1^{ab}	4.4^{abc}	3.7^{c}	5.1^{ab}	4.2^{bc}	4.2^{bc}	5.3^{ab}	5.6^{a}	5.2^{ab}
Crunchiness	84.45***	4.5^{a}	3.0^{b}	4.20*	3.6^{b}	3.9^{a}	3.74	26.23***	5.10***	3.1^{def}	3.0^{ef}	4.2^{bcd}	3.2^{def}	3.0^{ef}	2.5^{f}	3.3^{def}	3.8^{cde}	6.7^{a}	4.9^{b}	4.8^{bc}	2.5^{f}
Juiciness	7.36**	6.2^{a}	5.7^{b}	25.33***	6.3^{a}	5.5^{b}	4.32*	8.61***	3.19***	5.6^{cd}	5.2^{cd}	6.8^{ab}	5.2^{cd}	4.5^{d}	6.0^{abc}	7.2^{a}	6.1^{abc}	5.8^{bcd}	5.4^{cd}	6.0^{abc}	6.8^{ab}
Firmness	64.84***	4.2^{a}	3.0^{b}	5.65*	3.5^{b}	3.8^{a}	3.33	21.61***	3.92***	3.2^{cde}	3.1^{de}	4.0^{bcd}	3.0^{de}	3.2^{cde}	2.5^{e}	3.0^{de}	3.5^{b-e}	6.5^{a}	4.5^{b}	4.2^{bc}	2.6^{e}
Meltiness	40.72***	5.3^{b}	6.4^{a}	9.34**	6.1^{a}	5.6^{b}	6.32*	17.50***	3.53***	6.5^{a}	5.9^{abc}	5.8^{ab}	6.3^{abc}	6.3^{ab}	6.9^{a}	6.6^{a}	6.2^{abc}	2.7^{d}	5.0^{c}	5.1^{bc}	7.0^{a}
Mealiness	22.56***	2.0^{b}	2.9^{a}	66.92***	1.7^{b}	3.2^{a}	5.80*	7.82***	2.51**	3.0^{bcd}	3.2^{abc}	1.6^{e}	3.3^{ab}	4.3^{a}	3.0^{a-e}	1.8^{cde}	1.7^{de}	1.7^{cde}	2.2^{b-e}	1.9^{b-e}	2.1^{b-e}
Skin thickness	19.59***	4.8^{a}	4.0^{b}	1.86	4.6	4.3	0.02	5.44***	0.32	4.1^{bc}	3.9^{bc}	3.8^{c}	3.9^{bc}	3.7^{c}	4.1^{bc}	4.8^{abc}	4.2^{bc}	4.7^{bc}	6.3^{a}	5.4^{ab}	4.0^{bc}
Acid taste	15.20***	4.8^{a}	4.1^{b}	24.12***	4.9^{a}	4.0^{b}	3.27	6.24***	3.89***	3.5^{cd}	3.2^{d}	5.1^{ab}	4.1^{bcd}	3.7^{cd}	4.8^{abc}	5.3^{ab}	4.7^{abc}	5.7^{a}	4.7^{abc}	4.6^{abc}	3.9^{bcd}
Sweet taste	3.51	3.6	3.3	1.46	3.5	3.3	0.50	3.19***	2.40**	3.5^{ab}	3.3^{ab}	3.6^{ab}	3.0^{ab}	3.3^{ab}	2.6^{b}	3.7^{ab}	3.3^{ab}	2.8^{b}	4.0^{a}	4.0^{a}	4.1^{a}
Overall flavor	31.2***	5.0^{a}	4.2^{b}	16.03***	4.9^{a}	4.4^{b}	2.30	5.67***	1.55	4.4^{bcd}	4.1^{cd}	5.3^{ab}	4.1^{cd}	4.1^{cd}	3.7^{d}	5.1^{abc}	4.6^{a-d}	4.7^{abcd}	5.2^{abc}	5.5^{a}	4.7^{a-d}
Tomato flavor	20.15***	4.3^{a}	3.5^{b}	6.30*	4.1^{a}	3.6^{b}	0.36	3.53***	1.25	3.9^{abc}	3.4^{bc}	4.3^{abc}	3.4^{bc}	3.1^{bc}	2.9^{c}	4.4^{ab}	4.2^{abc}	3.8^{abc}	4.3^{abc}	5.0^{a}	3.9^{abc}

Aurea (AUR), Cauralina (CAU), Climberley (CLI), Garance (GAR), Hybrid Inra (HYB), Maillane (MAI), Marbonne (MNE), Marmande (MAR), Pera de Girona (PDG), Pomodoro di Sorrento (PDS), St. Pierre (STP), Valenciana (VAL).

Traditional varieties are in grey.

M= Modern varieties; T=Traditional varieties; S= soil; HS=soilless.

$^{(1)}$ F value; *p ≤ 0.05; **p ≤ 0.01; ***p ≤ 0.001

Within each testing condition, means with common superscript letters are not significantly different (p ≤ 0.05) according to the Tukey's (HSD) *post hoc* test. When a mean is followed by four or more letters, the range of letters is indicated.

Table S2. Analysis of variance and means of physicochemical parameters for varietal group (Modern/Traditional), growing condition (Soil/Soilless) and genotypes of tomato samples harvested at the red ripe stage in France (values are averages of three replicates).

Physicochemical parameter	Varietal group (V)			Growing Condition (GC)			(V)*(GC)	Genotype (G)	(G)*(GC)	Oxheart (Liguria)		Oxheart				Marmande		Round			
	(1)	M	T	(1)	S	HS	(1)	(1)	(1)	**AUR**	PDG	**CAU**	MAI	PDS	VAL	**MNE**	MAR	**CLI**	**GAR**	**HYB**	STP
FW (g)	4.12*	214^{b}	241^{a}	0.64	224	230	0.14	42.00***	4.64***	268^{a}	260^{a}	286^{a}	172^{bc}	303^{a}	309^{a}	312^{a}	201^{b}	134^{cd}	119^{d}	164^{bcd}	198^{b}
EC_L*	0.99	40.8	39.5	7.30**	41.7^{a}	38.5^{b}	4.52*	2.72**	3.72***	43.8^{ab}	34.9^{bc}	40.2^{abc}	41.9^{abc}	45.4^{a}	33.4^{c}	41.2^{abc}	40.6^{abc}	40.1^{abc}	40.3^{abc}	39.6^{abc}	40.1^{abc}
EC_a*	0.37	23.3	23.1	160.5***	24.5^{a}	21.8^{b}	<0.01	22.91***	5.30***	22.8^{bcd}	22.5^{cd}	22.7^{cd}	24.5^{ab}	25.0^{a}	21.6^{d}	24.1^{abc}	21.8^{d}	19.7^{e}	24.8^{a}	25.8^{a}	22.7^{cd}
EC_b*	4.87*	25.0^{b}	26.1^{a}	173.30***	27.1^{a}	23.9^{b}	1.03	42.83***	5.59***	24.8^{d}	27.4^{bc}	26.0^{cd}	29.3^{a}	20.3^{f}	28.8^{ab}	27.9^{ab}	25.8^{cd}	22.2^{ef}	24.6^{d}	24.5^{d}	24.2^{de}
FIRM (D.I.)	29.48***	61.3^{a}	52.8^{b}	19.95***	54.2^{b}	59.5^{a}	1.53	13.13***	3.69***	54.8^{bcd}	47.4^{d}	54.2^{bcd}	51.6^{bcd}	57.5^{c}	53.8^{bcd}	59.2^{b}	55.4^{bcd}	70.8^{a}	70.9^{a}	57.8^{bc}	48.3^{cd}
SSC (°Bx)	26.26***	5.37^{a}	4.93^{b}	190.80***	5.42^{a}	4.91^{b}	0.48	66.11***	2.89*	4.79^{fg}	4.87^{ef}	5.18^{cd}	4.98^{def}	5.14^{cde}	4.56^{g}	4.86^{ef}	4.86^{ef}	5.21^{cd}	6.48^{a}	5.68^{b}	5.35^{bc}
TA (g CA/100g)	14.88***	0.48^{a}	0.42^{b}	80.38***	0.51^{a}	0.40^{b}	6.02*	7.59***	4.83***	0.36^{e}	0.40^{cde}	0.50^{abc}	0.41^{cde}	0.38^{de}	0.47^{a-d}	0.47^{a-d}	0.46^{a-d}	0.52^{ab}	0.55^{a}	0.50^{abc}	0.41^{b-e}
Sucrose (g/kg)	0.17	0.38	0.39	4.72*	0.40^{a}	0.37^{b}	5.40*	3.51***	4.06***	0.39^{abc}	0.41^{ab}	0.38^{abc}	0.41^{ab}	0.36^{abc}	0.34^{bc}	0.38^{abc}	0.42^{ab}	0.33^{c}	0.43^{a}	0.40^{abc}	0.39^{abc}
Glucose (g/kg)	11.23**	16.2^{a}	14.9^{b}	37.80***	16.3^{a}	15.0^{b}	1.32	30.76***	5.21***	14.4^{de}	14.7^{cde}	16.1^{bc}	15.5^{bcd}	16.6^{b}	13.1^{e}	13.8^{e}	13.4^{e}	16.0^{bcd}	20.3^{a}	16.6^{b}	16.9^{b}
Fructose (g/kg)	9.90**	18.4^{a}	17.4^{b}	44.79***	18.6^{a}	17.3^{b}	1.74	22.35***	5.67***	17.0^{cd}	17.7^{bcd}	18.5^{b}	16.9^{cd}	18.3^{bc}	15.3^{e}	16.7^{de}	17.8^{bcd}	18.0^{bcd}	21.5^{a}	18.6^{b}	18.8^{b}
Citric acid (g/kg)	40.06***	5.6^{a}	4.8^{b}	387.66***	5.9^{a}	4.5^{b}	12.47***	37.60***	22.04***	4.6^{d}	3.8^{e}	6.1^{a}	5.1^{cd}	4.9^{d}	5.7^{ab}	5.5^{bc}	4.6^{d}	5.7^{ab}	5.9^{ab}	5.9^{ab}	4.6^{d}
Malic acid (g/kg)	21.67***	0.49^{b}	0.66^{a}	38.51***	0.63^{a}	0.53^{b}	0.17	47.93***	9.24***	0.38^{d}	0.92^{a}	0.48^{cd}	0.38^{d}	0.45^{cd}	0.50^{cd}	0.45^{cd}	0.94^{a}	0.65^{b}	0.53^{bc}	0.46^{cd}	0.81^{a}

Aurea (AUR), Cauralina (CAU), Climberley (CLI), Garance (GAR), Hybrid Inra (HYB), Maillane (MAI), Marbonne (MNE), Marmande (MAR), Pera de Girona (PDG), Pomodoro di Sorrento (PDS), St. Pierre (STP), Valenciana (VAL).

Traditional varieties are in grey.

FW= fruit weight, EC =external color, SSC= soluble solids content, TA= titratable acidity, FIRM = firmness (Durofel); °Bx = degrees Brix; D.I. = Durofel Index.

M= Modern varieties; T=Traditional varieties; S= soil; HS=soilless.

(1) F value; $^{*}p \leq 0.05$; $^{**}p \leq 0.01$; $^{***}p \leq 0.001$

Within each testing condition, means with common superscript letters are not significantly different ($p \leq 0.05$) according to the Tukey's (HSD) *post hoc* test. When a mean is followed by four or more letters, the range of letters is indicated.

Table S3. Contingency table of the frequencies of mentions by French consumers (n=100 eligible consumers) across all 14 tomato samples, evaluated at the red ripe stage, for individual terms of the Check-All-That-Apply (CATA) questions.

	Oxheart					Marmande				Round				
CATA Term	**CAU_HS**	**CAU_S**	MAI_HS	MAI_S	VAL_S	**MNE_HS**	**MNE_S**	MAR_HS	MAR_S	**CLI_HS**	**GAR_HS**	**GAR_S**	STP_HS	STP_S
Beautiful color ***	58^{bcd}	45^{ab}	52^{bc}	50^{bc}	51^{bc}	45^{ab}	54^{bc}	35^{a}	49^{bc}	69^{de}	68^{de}	76^{e}	73^{e}	60^{cd}
I like its appearance ***	51^{bcd}	48^{bc}	33^{a}	50^{bcd}	33^{a}	40^{ab}	40^{ab}	44^{ab}	52^{bcd}	63^{d}	62^{cd}	62^{cd}	61^{cd}	58^{cd}
Too big ***	38^{fgh}	27^{def}	24^{de}	6^{bc}	34^{efg}	40^{gh}	47^{h}	10^{c}	5^{abc}	1^{ab}	1^{ab}	11^{c}	0^{a}	20^{d}
I like its fleshy aspect ***	44^{e-h}	46^{fgh}	21^{a}	28^{abc}	42^{e-h}	54^{h}	50^{gh}	30^{a-d}	38^{a-e}	23^{ab}	23^{ab}	34^{b-f}	26^{abc}	39^{d-g}
I do not like its appearance ***	22^{bc}	26^{c}	29^{c}	22^{bc}	27^{c}	21^{bc}	24^{c}	25^{c}	19^{abc}	9^{a}	9^{a}	10^{a}	9^{a}	13^{ab}
Hollow fruit ***	2^{a}	3^{a}	61^{d}	27^{c}	5^{ab}	4^{a}	3^{a}	12^{b}	2^{a}	3^{a}	3^{a}	1^{a}	4^{ab}	3^{a}
Too small ***	1^{a}	3^{ab}	3^{ab}	4^{ab}	1^{ab}	0^{a}	0^{a}	7^{b}	5^{ab}	7^{b}	7^{b}	3^{ab}	27^{c}	0^{a}
Too much jelly and seeds ***	5^{a}	3^{a}	7^{ab}	8^{abc}	8^{abc}	9^{a-d}	10^{a-d}	15^{bcd}	8^{abc}	18^{d}	18^{d}	9^{abcd}	4^{a}	17^{cd}
Juicy ***	53^{de}	62^{ef}	21^{a}	36^{bc}	47^{cd}	68^{f}	68^{f}	50^{cde}	64^{ef}	42^{cd}	44^{cd}	28^{ab}	$64e^{f}$	68^{f}
Too thick skin ***	28^{b-e}	29^{b-f}	30^{b-f}	34^{c-f}	27^{bcd}	24^{abc}	21^{ab}	37^{def}	35^{c-f}	40^{ef}	41^{fg}	53^{g}	15^{a}	19^{ab}
Too firm ***	11^{b-e}	16^{cde}	8^{bcd}	9^{b-e}	7^{bc}	11^{b-e}	10^{b-e}	19^{e}	16^{de}	46^{f}	17^{de}	59^{g}	0^{a}	3^{ab}
Mealy ***	15^{bc}	8^{ab}	26^{c}	27^{c}	28^{c}	4^{a}	7^{ab}	16^{abc}	4^{a}	8^{ab}	11^{ab}	8^{ab}	15^{bc}	13^{ab}
Too soft ***	12^{cde}	6^{bc}	32^{f}	18^{de}	33^{f}	7^{bc}	10^{cd}	14^{cde}	21^{ef}	2^{ab}	7^{bc}	0^{a}	31^{f}	32^{f}
Too many seeds **	2^{a}	5^{ab}	16^{cd}	9^{a-d}	5^{ab}	14^{bcd}	15^{ab}	11^{bcd}	12^{bcd}	18^{d}	7^{abc}	10^{bcd}	11^{bcd}	12^{bcd}
Too much acid ***	8^{ab}	26^{d}	7^{ab}	12^{ab}	13^{b}	8^{ab}	14^{b}	8^{ab}	25^{cd}	10^{ab}	6^{ab}	28^{d}	4^{a}	15^{bc}
Not enough sweet ***	40^{a-e}	34^{abc}	44^{cde}	47^{de}	43^{b-e}	34^{a-d}	29^{a}	44^{cde}	34^{a-d}	39^{a-d}	29^{a}	53^{e}	30^{ab}	32^{abc}
Bland / no taste ***	36^{c-f}	18^{a}	56^{g}	45^{fg}	47^{fg}	28^{abc}	17^{a}	46^{fg}	24^{abc}	43^{efc}	24^{abc}	31^{b-e}	38^{def}	21^{ab}
Not pleasant after taste ***	11^{abc}	13^{abc}	17^{bcd}	15^{bcd}	26^{d}	10^{abc}	10^{abc}	13^{abc}	8^{ab}	10^{abc}	10^{abc}	19^{cd}	5^{a}	9^{ab}
Pleasant aroma ***	43^{bcd}	46^{bcd}	27^{a}	33^{ab}	25^{a}	54^{cde}	61^{e}	40^{bc}	48^{cde}	41^{bcd}	55^{de}	33^{ab}	50^{cde}	54^{cde}
Aromatic / strong aroma ***	19^{bc}	33^{ef}	12^{ab}	19^{bc}	18^{bc}	36^{f}	33^{f}	7^{a}	21^{b-e}	15^{ab}	30^{cdef}	18^{bc}	20^{bcd}	31^{def}

Aurea (AUR), Cauralina (CAU), Climberley (CLI), Garance (GAR), HybInra (HYB), Maillane (MAI), Marbonne (MNE), Marmande (MAR), Pera de Girona (PDG), Pomodoro di Sorrento (PDS), St. Pierre (STP), Valenciana (VAL). S= soil; HS=soilless. Traditional varieties are in grey.

Values within a row with common letters are not significantly different ($p \leq 0.05$). Multiple pairwise comparisons based on McNemar's test. When a mean is followed by four or more letters, the range of letters is indicated.

Asterisks indicate significant differences according to Cochran's Q test at *$p \leq 0.05$, **$p \leq 0.01$, ***$p \leq 0.001$, while ns indicates non-significant differences between samples.

Table S4. Analysis of variance and means of sensory descriptors for varietal group (Modern/Traditional), growing condition (Greenhouse/Open Field) and genotypes of tomato samples harvested at the red ripe stage in Spain (scale from 0 to 10, semi-structured continuous; values are averages of two replicates).

	Varietal group (V)			Growing Condition (GC)			(V)*(GC)	Genotype (G)	(G)*(GC)	Oxheart (Liguria)		Oxheart					Marmande			
Sensory descriptor	[(1)]	M	T	[(1)]	GH	OF	[(1)]	[(1)]	[(1)]	**AUR**	PDG	**CAU**	MAI	VAL	**TLP**	PDS	**MNE**	MAR	**MLD**	MUC
Overall odor	0.07	6.0	6.0	5.72^{*}	6.2^{a}	5.8^{b}	0.86	2.25^{*}	1.21	5.6^{b}	5.9^{ab}	5.8^{ab}	6.0^{ab}	5.6^{b}	6.0^{ab}	6.9^{a}	6.4^{ab}	5.7^{ab}	6.2^{ab}	6.1^{ab}
Acid taste	0.28	5.5	5.4	6.06^{*}	5.6^{a}	5.2^{b}	1.40	4.34^{***}	1.64	4.8^{b}	4.7^{b}	6.1^{a}	5.4^{ab}	6.3^{a}	5.5^{ab}	4.8^{b}	5.6^{ab}	6.1^{a}	5.2^{ab}	4.8^{b}
Sweet taste	8.86^{**}	5.6^{a}	5.0^{b}	3.59	5.1	5.5	2.33	5.77^{***}	3.46^{***}	5.4^{a}	5.8^{a}	5.8^{a}	5.4^{a}	5.1^{ab}	5.8^{a}	5.8^{a}	5.2^{ab}	4.1^{b}	5.6^{a}	3.9^{b}
Overall flavor	15.80^{***}	6.5^{a}	5.8^{b}	0.18	6.1	6.1	0.45	4.48^{***}	2.31^{*}	5.9^{ab}	6.0^{ab}	6.8^{a}	6.0^{ab}	5.8^{ab}	6.7^{a}	6.5^{a}	6.6^{a}	5.6^{ab}	6.3^{a}	4.8^{b}
Tomato flavor	11.24^{***}	5.8^{a}	5.2^{b}	1.61	5.4	5.7	0.51	6.55^{***}	2.41^{**}	5.4^{abc}	5.7^{abc}	6.4^{ab}	5.4^{abc}	5.1^{bcd}	6.1^{ab}	6.5^{a}	5.5^{abc}	4.6^{cd}	5.7^{abc}	3.8^{d}
Juiciness	0.53	6.4	6.3	3.81	6.5	6.2	1.26	3.38^{***}	1.5	6.3^{abc}	6.5^{abc}	6.5^{abc}	5.7^{c}	6.7^{abc}	6.3^{abc}	5.9^{bc}	6.9^{ab}	7.3^{a}	6.1^{abc}	5.8^{bc}
Firmness	2.33	5.1	4.8	7.62^{**}	5.2^{a}	4.7^{b}	0.07	3.34^{***}	1.2	5.0^{abc}	4.8^{abc}	5.3^{ab}	5.6^{a}	3.8^{c}	4.9^{abc}	3.9^{bc}	4.7^{bc}	5.1^{abc}	5.6^{a}	5.3^{ab}
Mealiness	1.27	2.8	3.0	9.18^{**}	2.7^{b}	3.2^{a}	3.90^{*}	4.30^{***}	2.90^{**}	2.7^{abcd}	2.0^{d}	2.3^{bcd}	2.8^{a-d}	3.8^{ab}	2.4^{bcd}	3.0^{a-d}	2.6^{bcd}	2.1^{cd}	3.7^{abc}	4.4^{a}
Skin thickness	0.77	6.3	6.5	3.45	6.2	6.6	0.97	3.67^{***}	0.43	5.3^{c}	5.6^{bc}	6.8^{abc}	6.6^{abc}	7.5^{a}	5.9^{abc}	6.1^{abc}	6.2^{abc}	6.9^{abc}	7.2^{ab}	6.1^{abc}

Aurea (AUR), Cauralina (CAU), Maillane (MAI), Marmande (MAR), Marmalindo (MLD), Marbonne (MNE), Muchamiel (MUC), Pera de Girona (PDG), Pomodoro di Sorrento (PDS), Toi Ls Pan (TLP), Valenciana (VAL).

Traditional varieties are in grey.

M= Modern varieties; T=Traditional varieties; GH= greenhouse; OF=open field.

[(1)] F value; $^{*}p \leq 0.05$; $^{**}p \leq 0.01$; $^{***}p \leq 0.001$

Within each testing condition, means with common superscript letters are not significantly different (p ≤ 0.05) according to the Tukey's (HSD) *post hoc* test.

Table S5. Analysis of variance and means of physicochemical parameters for varietal group (Modern/Traditional), growing condition (Greenhouse/Open Field) and genotypes of tomato samples harvested at the red ripe stage in Spain (values are averages of a minimum of two replicates).

Physicochemical parameter	Varietal group (V)			Growing Condition (GC)			(V)*(GC)	Genotype (G)	(G)*(GC)	Oxheart (Liguria)		Oxheart					Marmande			
	(1)	M	T	(1)	GH	OF	(1)	(1)	(1)	**AUR**	PDG	**CAU**	MAI	VAL	**TLP**	PDS	**MNE**	MAR	**MLD**	MUC
EC_L*	2.49	46.2	47.1	0.27	46.6	46.8	0.05	23.33***	5.34***	44.6^{e}	45.1^{de}	44.2^{e}	42.9^{e}	49.6^{b}	47.6^{bcd}	48.1^{bc}	45.6^{cde}	43.8^{e}	48.9^{b}	53.2^{a}
EC_a*	0.97	30.8	30.3	0.05	30.5	30.4	4.38*	13.90***	8.38***	33.2^{a}	30.8^{ab}	31.7^{ab}	32.1^{ab}	27.1^{cd}	29.6^{bc}	31.9^{ab}	32.6^{ab}	33.3^{a}	26.8^{cd}	25.6^{d}
EC_b*	7.88**	31.2^{b}	33.7^{a}	1.44	32.2	32.8	0.21	40.68***	3.27***	31.1^{de}	30.7^{e}	30.2^{e}	34.6^{bcd}	34.9^{bc}	23.3^{f}	26.3^{f}	33.8^{b-e}	32.2^{cde}	37.5^{b}	43.3^{a}
FIRM (D.I)	<.01	64.6	64.7	80.03***	61.6^{b}	67.9^{a}	14.85***	37.14***	8.44***	60.4^{d}	62.1^{cd}	62.0^{cd}	58.1^{d}	60.2^{d}	66.9^{bc}	67.2^{bc}	65.8^{bc}	57.8^{d}	69.0^{b}	82.7^{a}
SSC (°Bx)	15.20***	5.7^{a}	5.1^{b}	4.14*	5.2^{b}	5.5^{a}	5.02*	3.57***	1.65	5.43^{ab}	4.85^{b}	5.94^{a}	5.73^{ab}	5.14^{ab}	5.47^{ab}	5.23^{ab}	5.95^{a}	4.80^{b}	5.55^{ab}	4.65^{b}
TA (g CA/100g)	10.18**	0.32^{a}	0.26^{b}	0.10	0.29	0.29	<0.01	8.00***	1.48	0.27^{abc}	0.21^{c}	0.34^{ab}	0.26^{bc}	0.33^{ab}	0.27^{bc}	0.28^{abc}	0.34^{b}	0.30^{abc}	0.37^{a}	0.21^{c}
pH	6.26*	4.3^{b}	4.4^{a}	2.25	4.4	4.3	0.08	15.85***	1.70	4.45^{a}	4.47^{a}	4.26^{bcd}	4.45^{a}	4.37^{abc}	4.43^{a}	4.43^{a}	4.27^{bcd}	4.24cd	4.18^{d}	4.40^{ab}
DM (%)	5.40*	6.6^{a}	6.2^{b}	3.05	6.2	6.4	0.93	11.45***	2.64*	6.06^{cd}	6.25^{bcd}	7.11^{a}	6.50^{abc}	6.53^{abc}	6.34^{a-d}	6.59^{abc}	6.47^{abc}	5.74^{de}	6.82^{ab}	5.18^{e}

Aurea (AUR), Cauralina (CAU), Maillane (MAI), Marmande (MAR), Marmalindo (MLD), Marbonne (MNE), Muchamiel (MUC), Pera de Girona (PDG), Pomodoro di Sorrento (PDS), Toi Ls Pan (TLP), Valenciana (VAL).

Traditional varieties are in grey.

SSC= Soluble solids content, EC =external color, DM = Dry matter, TA= Titratable acidity, CA= Citric acid, FIRM = Firmness (Durofel); °Bx = degrees Brix; D.I. = Durofel Index.

M=Modern varieties; T=Traditional varieties; GH= greenhouse; OF=open field.

(1) F value; $^{*}p \leq 0.05$; $^{**}p \leq 0.01$; $^{***}p \leq 0.001$

Within each testing condition, means with common superscript letters are not significantly different ($p \leq 0.05$) according to the Tukey's (HSD) *post hoc* test. When a mean is followed by four or more letters, the range of letters is indicated.

Table S6. Contingency table of the frequencies of mentions by Spanish consumers (n= 115 eligible consumers) across all 16 tomato samples, evaluated at the red ripe stage, for individual terms of the CATA questions.

	Oxheart (Liguria)		Oxheart							Marmande						
CATA Term	**AUR_GH**	PDG_GH	**CAU_GH**	**CAU_OF**	MAI_GH	MAI_OF	VAL_GH	VAL-OF	PDS_GH	**MNE_GH**	**MNE_OF**	MAR_GH	MAR_OF	**MLD_GH**	**MLD_OF**	MUC_GH
Beautiful color ***	60^{cde}	36^{ab}	64^{cde}	66^{cde}	45^{abc}	49^{bc}	64^{cde}	61^{cde}	65^{cde}	73^{de}	80^{e}	47^{bc}	52^{bcd}	59^{b-e}	62^{cde}	22^{a}
I like its appearance ***	55^{a-e}	41^{ab}	63^{b-e}	63^{b-e}	47^{abc}	45^{abc}	49^{a-d}	68^{cde}	42^{ab}	62^{b-e}	66^{b-e}	48^{abc}	54^{a-d}	80^{e}	74^{de}	31^{a}
Too big ***	19^{d}	10^{a-d}	12^{a-d}	6^{a-d}	4^{abc}	1^{ab}	17^{cd}	10^{a-d}	34^{e}	10^{a-d}	7^{a-d}	6^{a-d}	0^{a}	7^{a-d}	3^{abc}	15^{bcd}
I like its fleshy aspect ***	41^{b-e}	22^{ab}	60^{e}	49^{de}	$32a^{bcd}$	22^{ab}	47^{cde}	41^{b-e}	51^{de}	44^{b-e}	51^{de}	37^{a-e}	24^{abc}	38^{a-e}	42^{b-e}	16^{a}
I do not like its appearance ***	24^{bc}	15^{abc}	9^{ab}	13^{ab}	32^{cd}	23^{abc}	17^{abc}	14^{abc}	20^{abc}	14^{abc}	7^{ab}	23^{abc}	18^{abc}	5^{a}	8^{ab}	47^{d}
Hollow fruit ***	7^{a}	58^{d}	3^{a}	3^{a}	23^{b}	38^{c}	0^{a}	1^{a}	4^{a}	1^{a}	1^{a}	1^{a}	6^{a}	1^{a}	1^{a}	1^{a}
Too small ***	3^{a}	3^{a}	3^{a}	3^{a}	6^{ab}	9^{ab}	0^{a}	1^{a}	0^{a}	3^{a}	3^{a}	6^{ab}	13^{b}	2^{a}	4^{ab}	3^{a}
Too much jelly and seeds ***	6^{a}	8^{ab}	2^{a}	3^{a}	6^{a}	5^{a}	6^{a}	4^{a}	2^{a}	7^{ab}	10^{ab}	10^{ab}	18^{b}	7^{ab}	7^{ab}	6^{a}
Juicy ***	64^{e}	38^{a-d}	70^{e}	48^{a-e}	47^{a-e}	30^{ab}	52^{b-e}	35^{abc}	51^{b-e}	54^{b-e}	68^{e}	55^{cde}	62^{d-e}	54^{b-e}	50^{b-e}	25^{a}
Too thick skin ***	22^{a}	18^{a}	32^{ab}	22^{a}	34^{ab}	58^{c}	39^{abc}	55^{bc}	19^{a}	40^{abc}	35^{abc}	41^{abc}	38^{abc}	53^{bc}	48^{bc}	49^{bc}
Too firm ***	7^{a}	2^{a}	11^{ab}	16^{ab}	12^{ab}	15^{ab}	4^{a}	7^{a}	13^{ab}	17^{ab}	5^{a}	11^{ab}	10^{ab}	17^{ab}	24^{b}	42^{c}
Mealy ***	8^{ab}	14^{a-d}	10^{ab}	15^{a-d}	9^{ab}	28^{cd}	17^{a-d}	23^{bcd}	25^{bcd}	4^{a}	11^{abc}	15^{a-d}	14^{a-d}	12^{abc}	13^{abc}	31^{d}
Too soft ***	13^{a-d}	29^{de}	5^{ab}	11^{bcd}	12^{abc}	7^{abc}	34^{e}	23^{cde}	13^{a-d}	8^{abc}	20^{b-e}	15^{a-d}	18^{b-e}	1^{a}	4^{ab}	8^{abc}
Too many seeds ***	3^{a}	5^{ab}	3^{a}	1^{a}	7^{ab}	4^{ab}	5^{ab}	7^{ab}	0^{a}	3^{a}	6^{ab}	10^{ab}	14^{b}	5^{ab}	5^{ab}	10^{ab}
Sweet ***	62^{d}	46^{a-d}	58^{cd}	50^{cde}	51^{bcd}	42^{a-d}	46^{a-d}	41^{a-d}	55^{bcd}	50^{bcd}	53^{bcd}	32^{ab}	35^{abc}	52^{bcd}	66^{d}	23^{a}
Too much acid ***	3^{a}	4^{ab}	15^{abc}	13^{abc}	10^{abc}	11^{abc}	22^{c}	19^{bc}	2^{a}	23^{c}	16^{abc}	13^{abc}	11^{abc}	15^{abc}	6^{ab}	12^{abc}
Not enough sweet ***	30^{a}	37^{ab}	32^{a}	43^{ab}	39^{ab}	34^{ab}	30^{a}	40^{ab}	28^{a}	34^{ab}	37^{ab}	51^{ab}	47^{ab}	36^{ab}	31^{a}	56^{b}
Bland / no taste ***	26^{a-e}	42^{c-f}	10^{a}	27^{a-f}	35^{b-f}	43^{def}	23^{a-d}	26^{a-e}	19^{ab}	20^{abc}	14^{ab}	46^{ef}	49^{fg}	25^{a-e}	22^{a-d}	70^{g}
Not pleasant aftertaste ***	13^{abc}	20^{bc}	6^{ab}	10^{abc}	12^{abc}	16^{abc}	17^{abc}	17^{abc}	8^{ab}	10^{abc}	12^{abc}	12^{abc}	7^{ab}	3^{a}	6^{ab}	26^{c}
Aromatic /strong aroma ***	40^{d}	18^{abc}	32^{cd}	24^{a-d}	22^{a-d}	16^{abc}	14^{abc}	16^{abc}	29^{bcd}	15^{abc}	20^{abc}	11^{ab}	17^{abc}	23^{a-d}	23^{a-d}	7^{a}

Aurea (AUR), Cauralina (CAU), Maillane (MAI), Marmande (MAR), Marmalindo (MLD), Marbonne (MNE), Muchamiel (MUC), Pera de Girona (PDG), Pomodoro di Sorrento (PDS), Valenciana (VAL). GH = greenhouse; OF = open field.

Traditional varieties are in grey.

Values within a row with common letters are not significantly different ($p < 0.05$) Multiple pairwise comparisons based on McNemar's test. When a mean is followed by four or more letters, the range of letters is indicated.

Asterisks indicates significant differences according to Cochran's Q test at *p ≤ 0.05, **p ≤ 0.01, ***p ≤ 0.001, while ns indicates non-significant differences between samples.

Table S7. Analysis of variance and means of sensory descriptors for varietal group (Modern/Traditional) and genotypes of tomato samples harvested at the early ripe stage (ER) in Italy (scale from 0 to 9, continuous; values are averages of two replicates).

	Varietal group (V)			Genotype (G)	Oxheart (Liguria)		Oxheart							Marmande		
Sensory descriptor	(1)	M	T	(1)	**AUR**	PDG	**CAU**	MAI	VAL	**RDS**	**TLP**	PDS	PDS2	**MNE**	MAR	MUC
External color	6.26^{*}	5.3^{a}	4.6^{b}	2.00^{*}	5.7	4.1	4.9	5.3	3.8	4.8	4.8	4.3	5.6	5.5	5.1	4.5
Seeds	<0.01	5.1	5.1	4.62^{***}	5.6^{ab}	4.8^{abc}	5.1^{abc}	3.3^{bc}	5.0^{abc}	2.6^{c}	4.6^{abc}	3.3^{bc}	4.7^{abc}	5.7^{ab}	7.3^{a}	5.7^{ab}
Pulpiness	3.23	6.7	6.3	5.24^{***}	6.5^{a-d}	6.1^{cd}	6.7^{a-d}	7.5^{ab}	6.2^{bcd}	7.2^{abc}	6.8^{a-d}	7.7^{a}	7.2^{abc}	6.7^{a-d}	5.6^{d}	6.1^{cd}
Overall odor	1.21	6.9	6.7	1.30	6.9	6.6	6.7	6.1	6.7	7.0	6.7	7.3	7.1	7.1	6.7	6.8
Tomato odor	0.72	6.3	6.1	0.73	6.2	6.0	6.3	5.9	6.0	6.4	6.0	6.5	6.6	6.3	6.0	6.0
Sweet taste	2.99	4.0	3.6	4.6^{***}	4.4^{abc}	3.7^{a-d}	3.5^{a-d}	3.7^{a-d}	2.9^{d}	4.7^{ab}	4.4^{abc}	4.8^{a}	4.6^{b}	3.6^{a-d}	3.3^{bcd}	3.1^{cd}
Acid taste	1.81	3.4	3.0	4.30^{***}	3.2^{abc}	2.8^{abc}	3.8^{a}	3.1^{abc}	4.0^{a}	2.6^{abc}	3.0^{abc}	1.4^{c}	2.0^{bc}	3.7^{ab}	3.7^{ab}	3.3^{ab}
Overall flavor	6.58^{*}	6.9^{a}	6.4^{b}	1.33	7.0	6.0	6.7	6.2	6.7	6.9	6.9	7.1	7.0	7.0	6.5	6.2
Tomato flavor	6.41^{*}	6.3^{a}	5.7^{b}	1.90^{*}	6.4	5.5	6.1	5.6	5.6	6.2	6.5	6.7	6.5	6.2	5.7	5.2
Firmness	3.95^{*}	4.4^{b}	4.9^{a}	1.41	4.1	5.1	5.3	4.8	4.6	4.6	4.6	4.3	4.4	4.4	5.0	4.9
Juiciness	16.57^{***}	7.1^{a}	6.1^{b}	2.79^{**}	7.3^{a}	5.5^{b}	6.7^{ab}	6.1^{ab}	6.5^{ab}	5.9^{ab}	6.8^{ab}	6.3^{ab}	6.4^{ab}	7.6^{a}	7.0^{ab}	6.2^{ab}
Mealiness	1.66	2.3	2.7	1.46	2.8	3.3	2.4	3.1	2.7	3.0	1.8	3.4	3.2	1.6	1.7	2.1
Skin thickness	0.15	4.8	5.0	0.31	4.6	5.0	5.3	4.8	5.5	4.8	4.6	5.0	4.9	5.0	5.0	4.7

Aurea (AUR), Cauralina (CAU), Maillane (MAI), Marmande (MAR), Marbonne (MNE), Muchamiel (MUC), Pera de Girona (PDG), Pomodoro di Sorrento (PDS), Pomodoro di Sorrento (Landrace 2) (PDS2), Rosa di Sorrento (RDS), Toi Ls Pan (TLP), Valenciana (VAL). Traditional varieties are in grey.

M= Modern varieties; T=Traditional varieties.

(1) F value; $^{*}p \leq 0.05$; $^{**}p \leq 0.01$; $^{***}p \leq 0.001$

Within each testing condition, means with common superscript letters are not significantly different ($p \leq 0.05$) according to the Tukey's (HSD) *post hoc* test. When a mean is followed by four or more letters, the range of letters is indicated.

Table S8. Analysis of variance and means of physicochemical parameters for varietal group (Modern/Traditional) and genotypes of tomato samples harvested at the early ripe (ER) stage in Italy (values are averages of four replicates).

	Varietal group (V)			Genotype (G)	Oxheart (Liguria)		Oxheart							Marmande		
Physicochemical parameter	[(1)]	M	T	[(1)]	**AUR**	PDG	**CAU**	MAI	VAL	**RDS**	**TLP**	PDS	PDS2	**MNE**	MAR	MUC
FW (g)	0.08	229	225	8.96^{***}	181^{d}	223^{bcd}	169^{d}	218^{cd}	301^{a}	260^{abc}	293^{ab}	272^{abc}	241^{a-d}	266^{abc}	176^{d}	213^{cd}
EC_L*	0.01	48.8	48.9	3.89^{***}	51.5^{a}	48.4^{ab}	43.9^{b}	49.2^{a}	49.2^{a}	49.0^{a}	48.1^{ab}	51.5^{a}	50.5^{a}	48.0^{ab}	47.4^{ab}	48.94^{a}
EC_a*	4.05	9.5	5.6	1.68	10.9	7.7	7.5	10.1	8.4	9.2	12.0	1.2	7.5	8.0	4.3	0.1
EC_b*	2.95	27.1	28.8	14.4^{***}	29.1^{ab}	28.4^{bc}	24.3^{cde}	29.7^{ab}	29.41^{ab}	25.5^{bcd}	20.51^{e}	28.4^{bc}	23.3^{de}	28.8^{ab}	27.3^{bcd}	33.2^{a}
FIRM (D.I.)	0.61	78.1	75.7	0.89	81.4	70.6	73.7	72.0	74.3	80.1	73.9	83.6	77.7	77.1	75.4	83.6
SSC (°Bx)	2.50	4.7	4.6	10.90^{***}	4.7^{abc}	4.7^{abc}	4.6^{abc}	4.5^{bc}	4.7^{abc}	5.0^{a}	4.7^{abc}	5.0^{ab}	4.9^{abc}	4.6^{abc}	4.5^{c}	3.9^{d}
TA (g CA/100g)	8.45^{***}	0.60^{a}	0.53^{b}	14.72^{***}	0.54^{d}	0.49^{de}	0.65^{ab}	0.54^{cd}	0.65^{ab}	0.53^{d}	0.57^{bcd}	0.50^{de}	0.57^{bcd}	0.69^{a}	0.64^{abc}	0.41^{e}
pH	20.88^{***}	4.07^{b}	4.16^{a}	24.53^{***}	4.13^{bcd}	4.19^{ab}	4.04^{ef}	4.20^{ab}	4.08^{cde}	4.16^{abc}	4.08^{de}	4.20^{ab}	4.12^{cd}	3.98^{f}	4.06^{de}	4.22^{a}

Aurea (AUR), Cauralina (CAU), Maillane (MAI), Marmande (MAR), Marmalindo (MLD), Marbonne (MNE), Muchamiel (MUC), Pera de Girona (PDG), Pomodoro di Sorrento (PDS), Pomodoro di Sorrento (Landrace 2) (PDS2), Rosa di Sorrento (RDS), Toi Ls Pan (TLP), Valenciana (VAL). Traditional varieties are in grey.

FW= fruit weight, SSC= Soluble solids content, EC =external color, TA= titratable acidity, FIRM = firmness (Durofel); °Bx = degrees Brix; D.I. = Durofel Index.

M= Modern variety; T=Traditional variety.

[(1)] F value; $^{*}p \leq 0.05$; $^{**}p \leq 0.01$; $^{***}p \leq 0.001$

Within each testing condition, means with common superscript letters are not significantly different ($p \leq 0.05$) according to the Tukey's (HSD) *post hoc* test. When a mean is followed by four or more letters, the range of letters is indicated.

Table S9. Analysis of variance and mean of sensory descriptors for varietal group (Modern/Traditional) and genotypes of tomato samples harvested at the red ripe stage (RR) in Italy (scale from 0 to 9, continuous; values are averages of two replicates).

	Varietal group (V)			Genotype (G)	Oxheart (Liguria)		Oxheart							Marmande		
Sensory descriptor	(1)	M	T	(1)	**AUR**	PDG	**CAU**	MAI	VAL	**RDS**	**TLP**	PDS	PDS2	**MNE**	MAR	MUC
External color	0.54	7.6	7.4	2.52^{**}	7.8^{ab}	7.8^{a}	6.7^{ab}	7.4^{ab}	7.5^{ab}	7.1^{ab}	7.8^{ab}	6.4^{b}	7.3^{ab}	7.7^{ab}	7.6^{ab}	6.9^{ab}
Seeds	0.02	4.9	4.8	4.87^{***}	5.0^{abc}	5.3^{abc}	4.6^{abc}	3.24^{c}	4.6^{abc}	2.5^{c}	3.02^{c}	2.6^{c}	3.5^{bc}	6.2^{ab}	6.7a	4.5^{abc}
Pulpiness	0.08	6.8	6.7	1.78	6.6	6.6	6.9	7.2	6.4	7.5	7.5	7.3	7.4	6.4	5.9	7.0
Overall odor	6.18^{*}	7.5^{a}	7.2^{b}	2.02^{*}	7.1	7.2	7.3	7.0	7.0	7.7	7.8	7.0	7.2	7.7	7.1	7.4
Tomato odor	3.74	7.2	6.8	1.43	6.7	6.9	7.0	6.8	6.7	7.4	7.3	6.9	6.9	7.5	6.6	7.0
Sweet taste	4.58^{*}	5.3^{a}	4.9^{b}	4.24^{***}	5.5^{ab}	5.3^{ab}	5.4^{ab}	4.9^{ab}	4.7^{ab}	5.6^{a}	6.0^{a}	5.6^{a}	5.6^{a}	4.7^{ab}	4.3^{b}	4.3^{b}
Acid taste	0.56	2.0	1.8	4.37^{***}	1.7^{ab}	1.6^{b}	1.1^{b}	1.9^{ab}	2.1^{ab}	1.1^{b}	2.0^{ab}	1.0^{b}	1.1^{b}	2.8^{a}	2.3^{b}	2.3^{ab}
Overall flavor	4.87^{*}	7.6^{a}	7.1^{b}	0.85	7.5	7.2	7.5	7.1	7.0	7.7	7.8	7.2	7.4	7.6	6.9	6.8
Tomato flavor	3.90	7.3	6.8	0.81	7.1	6.8	7.3	6.8	6.6	7.3	7.7	7.0	7.2	7.4	6.6	6.5
Firmness	0.17	4.3	4.2	2.98^{**}	4.6^{b}	3.3^{b}	4.4^{ab}	4.9^{a}	4.1^{ab}	4.2^{ab}	3.9^{ab}	5.3^{a}	4.2^{ab}	4.1^{ab}	4.8^{a}	4.6^{ab}
Juiciness	2.78	7.6	7.2	0.64	7.4	7.2	7.6	6.9	7.3	7.3	7.6	7.2	7.2	7.8	7.5	7.2
Mealiness	0.07	1.4	1.5	0.22	1.0	1.6	1.3	1.3	1.7	1.9	1.5	1.3	1.2	1.5	1.2	1.5
Skin thickness	1.76	5.2	4.7	0.61	5.0	4.1	5.1	4.9	4.9	4.6	5.0	5.2	4.5	5.5	5.1	5.3

Aurea (AUR), Cauralina (CAU), Maillane (MAI), Marmande (MAR), Marbonne (MNE), Muchamiel (MUC), Pera de Girona (PDG), Pomodoro di Sorrento (PDS), Pomodoro di Sorrento (Landrace 2) (PDS2), Rosa Di Sorrento (RDS), Toi Ls Pan (TLP), Valenciana (VAL). Traditional varieties are in grey

M= Modern varieties; T=Traditional varieties

(1) F value; $^{*}p \leq 0.05$; $^{**}p \leq 0.01$; $^{***}p \leq 0.001$

Within each testing condition, means with common superscript letters are not significantly different ($p \leq 0.05$) according to the Tukey's (HSD) *post hoc* test.

Table S10. Analysis of variance and means of physicochemical parameters for varietal group (Modern/Traditional) and genotypes of tomato samples harvested at the red ripe stage (RR) in Italy (values are averages of four replicates).

	Varietal group (V)			Genotype (G)	Oxheart (Liguria)		Oxheart							Marmande		
Physicochemical parameter	[(1)]	M	T	[(1)]	**AUR**	PDG	**CAU**	MAI	VAL	**RDS**	**TLP**	PDS	PDS2	**MNE**	MAR	MUC
FW (g)	1.76	214	199	6.98***	183^{cd}	204^{bcd}	177^{cd}	210^{a-d}	249^{ab}	247^{ab}	272^{a}	207^{bcd}	201^{bcd}	228^{abc}	156^{d}	195^{bcd}
EC_L*	0.86	39.8	40.3	6.58***	39.9^{cd}	40.2^{bcd}	38.6^{d}	39.64^{cd}	43.0^{ab}	43.2^{a}	39.8^{cd}	42.0^{abc}	40.0^{cd}	38.9^{d}	38.9^{d}	40.5^{a-d}
EC_a*	22.21***	22.9^{a}	20.5^{b}	11.12***	23.1^{ab}	18.7^{d}	20.0^{cd}	23.8^{a}	20.7^{bcd}	22.0^{abc}	22.8^{ab}	22.7^{ab}	20.8^{bcd}	24.1^{a}	19.8^{cd}	22.1^{abc}
EC_b*	0.40	23.7	24.5	26.18***	25.3^{b}	25.2^{b}	24.2^{bc}	25.9^{ab}	29.77^{a}	20.5^{cd}	16.45^{e}	18.7^{de}	16.8^{de}	25.4^{b}	24.7^{b}	26.5^{ab}
FIRM (D.I.)	0.27	52.8	51.8	3.81**	49.1^{b}	46.1^{b}	51.5^{ab}	50.7^{b}	57.4^{ab}	62.9^{a}	51.4^{ab}	57.3^{ab}	56.4^{ab}	54.2^{ab}	49.6^{b}	58.0^{ab}
SSC (°Bx)	8.81**	5.5^{a}	5.2^{b}	6.37***	5.5^{a}	5.4^{a}	5.3^{a}	5.1^{a}	5.2^{a}	5.6^{a}	5.4^{a}	5.5^{a}	5.5^{a}	5.4^{a}	5.0^{a}	4.3^{b}
TA (g CA/100g)	37.01***	0.59^{a}	0.49^{b}	30.09***	0.51^{cd}	0.40^{e}	0.60^{b}	0.46^{de}	0.59^{b}	0.51^{cd}	0.56^{bc}	0.49^{cd}	0.48^{d}	0.71^{a}	0.60^{b}	0.56^{bc}
pH	24.82***	4.1^{b}	4.2^{a}	20.75***	4.20^{bcd}	4.29^{ab}	4.05^{fg}	4.33^{a}	4.17^{cde}	4.20^{bcd}	4.07^{efg}	4.25^{abc}	4.23^{bc}	3.98^{g}	4.29^{def}	4.07^{efg}

Aurea (AUR), Cauralina (CAU), Maillane (MAI), Marmande (MAR), Marmalindo (MLD), Marbonne (MNE), Muchamiel (MUC), Pera de Girona (PDG), Pomodoro di Sorrento (PDS), Pomodoro di Sorrento (Landrace 2) (PDS2), Rosa di Sorrento (RDS), Toi Ls Pan (TLP), Valenciana (VAL). Traditional varieties are in grey.

M= Modern variety; T=Traditional variety.

FW= fruit weight, SSC= Soluble solids content, EC =external color, TA= titratable acidity, FIRM = firmness (Durofel); °Bx = degrees Brix; D.I. = Durofel Index.

[(1)] F value; $^{*}p \leq 0.05$; $^{**}p \leq 0.01$; $^{***}p \leq 0.001$

Within each testing condition, means with common superscript letters are not significantly different ($p \leq 0.05$) according to the Tukey's (HSD) *post hoc* test. When a mean is followed by four or more letters, the range of letters is indicated.

Table S11. Contingency table of the frequencies of mentions by Italian consumers (n=107 eligible consumers) across all 12 tomato samples, evaluated at the early ripe (ER) stage, for individual terms of the CATA questions.

	Oxheart (Liguria)		Oxheart							Marmande		
CATA Term	**AUR**	PDG	**CAU**	MAI	VAL	**RDS**	**TLP**	PDS	PDS2	**MNE**	MAR	MUC
Beautiful color ***	70^{f}	55^{b-e}	70^{f}	59^{c-f}	41^{ab}	60^{c-f}	48^{abc}	66^{def}	53^{bcd}	69^{ef}	66^{def}	38^{a}
I like its appearance ***	62^{cde}	52^{a-d}	65^{de}	46^{ab}	44^{ab}	49^{abc}	52^{bcd}	70^{e}	52^{bcd}	61^{cde}	56^{b-e}	37^{a}
Too green ***	21^{a}	32^{abc}	26^{a}	33^{abc}	46^{cd}	39^{bcd}	53^{d}	26^{ab}	43^{cd}	33^{abc}	41^{cd}	75^{e}
Too big ***	20^{ab}	33^{cd}	12^{a}	41^{cd}	32^{bc}	42^{cd}	41^{cd}	45^{d}	40^{cd}	42^{cd}	9^{a}	19^{ab}
I like its fleshy aspect ns	70	61	67	66	55	61	59	70	63	73	58	57
Too red **	12^{b}	13^{bc}	9^{ab}	12^{b}	16^{bc}	12^{b}	8^{ab}	23^{c}	12^{b}	18^{bc}	8^{ab}	4^{a}
I do not like its appearance ns	51	51	57	53	56	50	44	44	49	47	42	53
Too pale color ***	39^{b-e}	44^{c-f}	35^{a-d}	47^{d-g}	55^{fg}	30^{abc}	60^{g}	30^{ab}	47^{d-g}	25^{a}	35^{a-d}	53^{efg}
Hollow fruit ***	9^{a}	39^{e}	10^{abc}	18^{a-d}	18^{a-d}	17^{a-d}	19^{abd}	23^{d}	19^{a-d}	9^{a}	18^{a-d}	19^{a-d}
Too small ***	20^{cd}	16^{bc}	31^{e}	16^{bc}	12^{abc}	7^{a}	10^{ab}	8^{ab}	15^{abc}	14^{abc}	40^{e}	28^{de}
Too much jelly and seeds ***	28^{bcd}	36^{de}	19^{ab}	13^{a}	31^{bcd}	25^{bcd}	23^{abc}	19^{ab}	22^{abc}	35^{cde}	47^{e}	34^{cde}
Juicy ***	79^{cd}	66^{bc}	78^{cd}	62^{ab}	77^{cd}	75^{bcd}	69^{bc}	82^{d}	71^{bcd}	80^{cd}	82^{d}	50^{a}
Too thick skin ***	31^{ab}	46^{cd}	24^{a}	38^{bc}	42^{bc}	41^{bc}	42^{bc}	33^{abc}	57^{de}	45^{bcd}	35^{abc}	62^{e}
Too firm ***	23^{ab}	20^{ab}	24^{abc}	28^{bcd}	29^{bcd}	21^{ab}	40^{d}	12^{a}	30^{bcd}	28^{bcd}	36^{cd}	64^{e}
Mealy ***	29^{abc}	37^{b-e}	25^{ab}	30^{a-d}	36^{bcd}	50^{e}	30^{a-d}	43^{de}	39^{cde}	34^{bcd}	19^{a}	27^{abc}
Too soft **	22^{abc}	32^{c}	21^{abc}	17^{ab}	33^{c}	28^{bc}	18^{ab}	30^{c}	29^{bc}	26^{bc}	26^{bc}	12^{a}
Too many seeds ***	24^{abc}	33^{c}	17^{a}	13^{a}	29^{bc}	22^{abc}	19^{ab}	14^{a}	19^{ab}	36^{cd}	49^{d}	30^{bc}
Sweet ***	58^{cd}	51^{bcd}	50^{bc}	52^{bcd}	39^{ab}	64^{d}	50^{bcd}	65^{d}	53^{bcd}	52^{bcd}	52^{bcd}	34^{a}
Too much acid ***	36^{bc}	40^{bc}	40^{bc}	30^{ab}	57^{d}	31^{ab}	31^{ab}	22^{a}	31^{ab}	45^{cd}	48^{cd}	47^{cd}
Not enough sweet ns	55	50	54	56	64	58	46	49	54	57	43	63
Bland / no taste **	33^{a}	50^{bc}	38^{ab}	34^{a}	43^{ab}	45^{ab}	45^{ab}	38^{ab}	40^{ab}	39^{ab}	36^{ab}	60^{c}
Not pleasant aftertaste ***	32^{abc}	37^{abc}	42^{b-e}	30^{ab}	55^{e}	34^{abc}	46^{cde}	25^{a}	42^{b-e}	38^{a-d}	41^{b-e}	52^{de}
Pleasant aroma ns	64	53	59	56	53	65	61	64	69	50	67	57
Aromatic/strong aroma *	54^{bc}	44^{abc}	47^{bc}	43^{ab}	49^{bc}	47^{bc}	52^{bc}	44^{abc}	48^{bc}	57^{bc}	58^{c}	33^{a}

Aurea (AUR), Cauralina (CAU), Maillane (MAI), Marmande (MAR), Marbonne (MNE), Muchamiel (MUC), Pera de Girona (PDG), Pomodoro di Sorrento (PDS), Pomodoro di Sorrento (Landrace 2) (PDS2), Rosa Di Sorrento (RDS), Toi Ls Pan (TLP), Valenciana (VAL). Traditional varieties are in grey.

Values within a row with common letters are not significantly different (p ≤ 0.05). Multiple pairwise comparisons based on McNemar's test. When a mean is followed by four or more letters, the range of letters is indicated. Asterisks indicate significant differences according to Cochran's Q test at *p ≤ 0.05, **p ≤ 0.01, ***p ≤ 0.001, while ns indicates non-significant differences between samples.

Table S12. Contingency table of the frequencies of mentions by Italian consumers (n=107 eligible consumers) across all 8 tomato samples, evaluated at the red ripe (RR) stage, for individual terms of the CATA questions.

	Oxheart (Liguria)		Oxheart				Marmande	
CATA Term	**AUR**	PDG	**CAU**	MAI	VAL	PDS	**MNE**	MAR
Beautiful color *	74^{abc}	74^{abc}	83^{c}	65^{ab}	72^{abc}	62^{a}	73^{abc}	77^{bc}
I like its appearance ns	66	66	71	58	61	59	68	66
Too green **	4^{abc}	6abc	1^{a}	2^{ab}	12c	10c	4^{abc}	7^{bc}
Too big ***	14^{abc}	16^{bc}	10^{ab}	34^{de}	21c	39e	24^{cd}	6^{a}
I like its fleshy appearance ns	68	67	71	66	66	65	72	60
Too red ***	60^{cd}	48^{a-d}	47^{abc}	60^{d}	40^{ab}	59^{cd}	53^{bcd}	38^{a}
I do not like its appearance ns	39	45	44	43	49	45	47	45
Too pale color *	4^{a}	14^{c}	6^{abc}	5^{ab}	13^{bc}	12^{abc}	9^{abc}	14^{c}
Hollow fruit ***	14^{ab}	37^{c}	11^{ab}	8^{a}	18^{ab}	21^{b}	10^{ab}	15^{ab}
Too small ***	28^{c}	14^{ab}	35^{c}	10^{a}	11^{a}	9^{a}	23^{bc}	53^{d}
Too much jelly and seeds ***	38^{cde}	46^{de}	19^{a}	25^{ab}	28^{abc}	33^{bcd}	39^{cde}	51^{e}
Juicy ns	97	96	92	93	88	87	97	96
Too thick skin ns	24	25	23	28	33	27	30	23
Too firm *	3^{ab}	3^{ab}	8^{b}	0^{a}	7^{b}	11^{b}	5^{ab}	4^{ab}
Mealy ns	39	35	31	37	42	36	29	26
Too soft ***	61^{bc}	74^{c}	51^{b}	67^{c}	44^{a}	51^{ab}	51^{ab}	48^{ab}
Too many seeds ***	35^{bc}	45^{cd}	24^{ab}	19^{a}	26^{ab}	32^{bc}	38^{bc}	55^{d}
Sweet ns	64	63	64	62	58	64	62	51
Too much acid ***	18^{a}	24^{abc}	36^{cd}	18^{a}	23^{ab}	19^{a}	43^{d}	35^{bcd}
Not enough sweet ns	48	47	39	43	40	43	44	51
Bland /no taste ns	34	36	22	41	33	32	28	33
Not pleasant aftertaste ns	29	35	33	32	30	32	38	30
Pleasant aromans	66	59	69	64	68	69	64	65
Aromatic/strong aroma ns	56	47	53	46	49	53	56	52

Aurea (AUR), Cauralina (CAU), Maillane (MAI), Marmande (MAR), Marbonne (MNE), Pera de Girona (PDG), Pomodoro di Sorrento (PDS), Valenciana (VAL). Traditional varieties are in grey.

Values within a row with common letters are not significantly different ($p \leq 0.05$). Multiple pairwise comparisons based on McNemar test. When a mean is followed by four or more letters, the range of letters is indicated. Asterisks indicate significant differences according to Cochran's Q test at *$p \leq 0.05$, **$p \leq 0.01$, ***$p \leq 0.001$, while ns indicates non-significant differences between samples.
